# Supplementary material for: Epidemiology, mortality, and health service use of local-level multimorbidity patterns in South Spain
Source: Nat Commun. 2023 Nov 24;14:7689. doi: 10.1038/s41467-023-43569-5 (PMC10673852; doi:10.1038/s41467-023-43569-5)
Supplement: Supplementary file 1 — Supplementary Information [file 41467_2023_43569_MOESM1_ESM.pdf]

Supplementary information

Oct 31, 2023

1. Supplementary Report, *Characterisation of multimorbidity patterns by local health area in South Spain: epidemiology and impact on the use of health services.*

In this report we will develop the methodology followed to obtain the multimorbidity patterns in the sample of the province of Cádiz. To do so, we first started with 1375068 individuals with medical records, which we transformed into 1142367 individuals in Cádiz. On them, we first analysed the prevalence of the 64 chronic conditions. In the following figure (Supplementary Figure 1) we can see this prevalence and also a list of all these conditions (the pathology "Other Cancer" englobes Head and neck cancer, Bronchus and lung cancer, Stomach cancer, Bone cancer, Skin Melanoma, Leukaemia, Liver Cancer, Pancreatic cancer, Kidney cancer, Renal pelvis cancer, Thyroid cancer, Bladder cancer, Immunoproliferative cancer, No Hodgkin Lymphoma, Hodgkin's disease, and Kaposi's sarcoma).

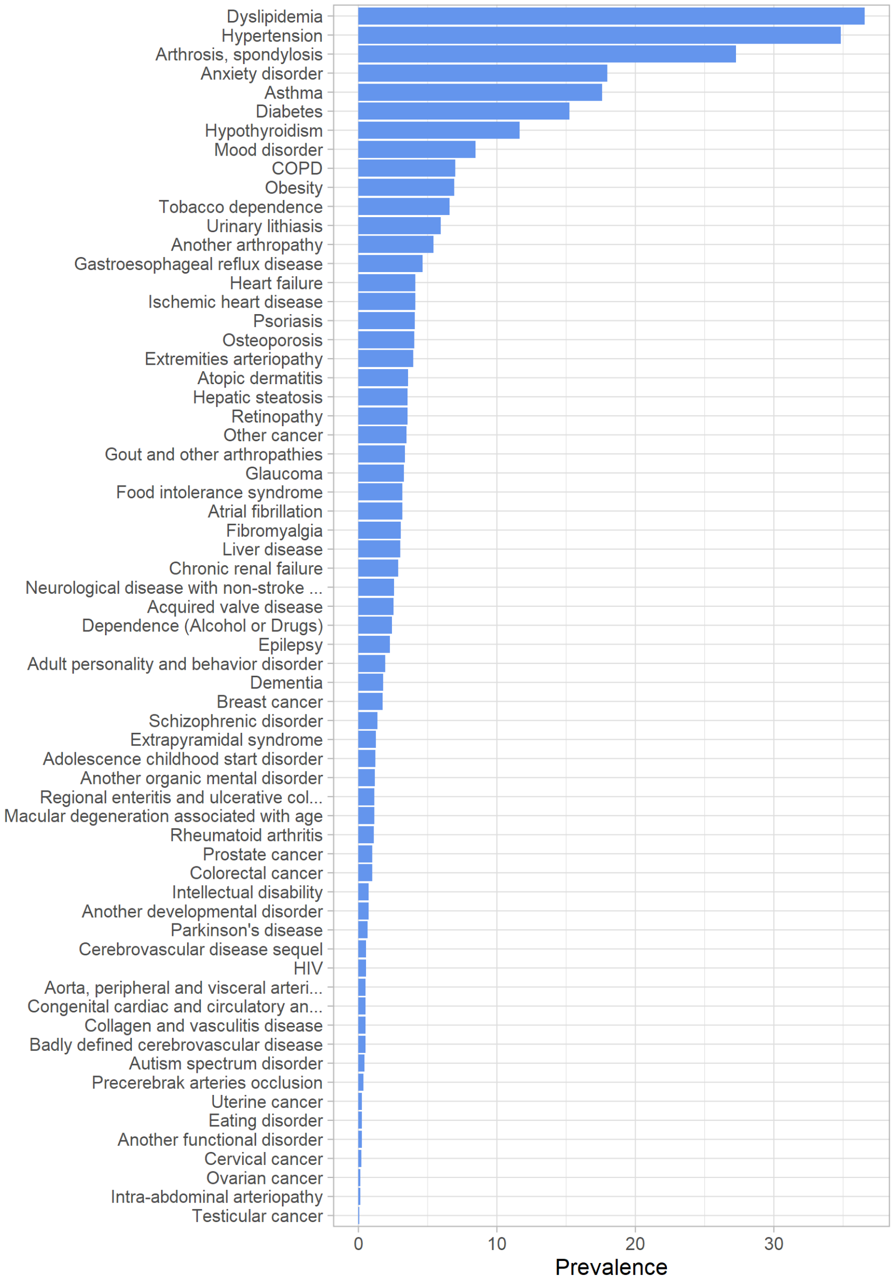

"

Supplementary Figure 1 | Chronic Conditions Prevalence. The bars show the overall prevalence in all the population of the 64 chronic conditions in the PHD database

With this 64 conditions, we stratified the database into groups according to sex and age (<16,16-44, 45-64, 65-79 and >79), obtaining 10 groups. In each stratum, we kept from the 64 chronic conditions those with

more than 1% prevalence, to simplify the analysis and to avoid spurious relationships. After that, we chose those individuals with multimorbidity (2 or more chronic conditions) in each stratum.

This generated 10 sex and age groups with the following sample sizes: 8047, 11789, 38130, 50817, 104398, 88420, 73326, 60129, 33700 and 20410. The total number of people with multimorbidity was 490130. In the following, we will describe the selection process of the latent classes in each stratum.

1) <16 and women

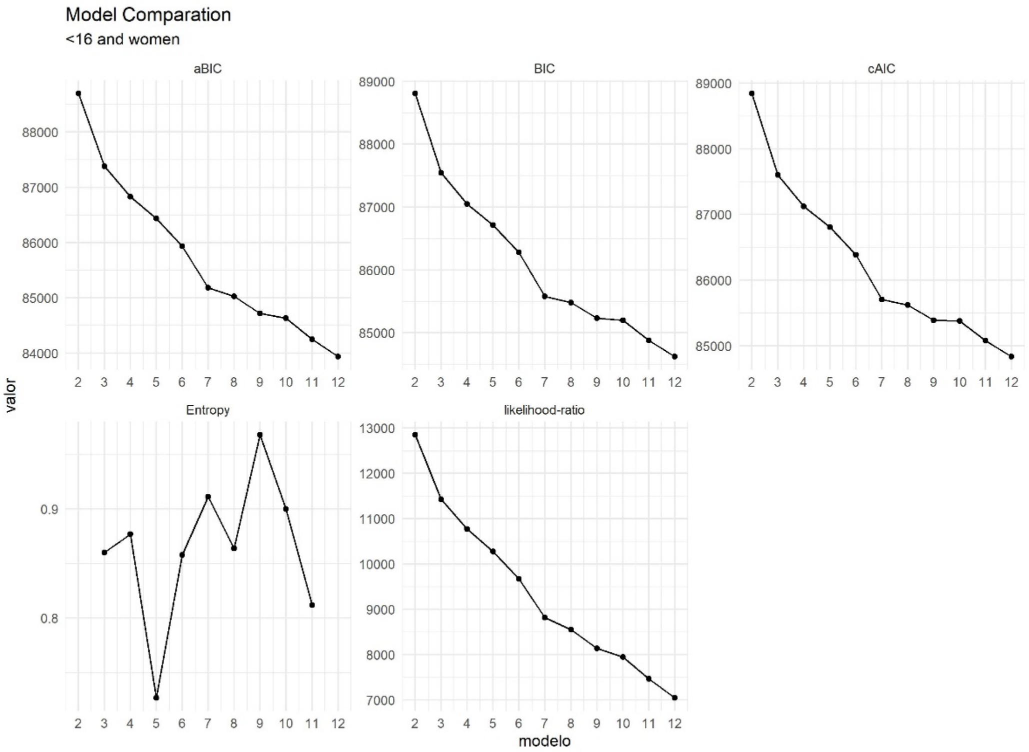

Supplementary Figure 2 | Godness-of-Fit Indices in the LCA Model with <16 and women. Looking at the lines, the chosen model is determined by trying to find the point where the slope starts to decrease the less

We can choose between the 7- and 8-class models (Supplementary Figure 2), which is where the decrease in GoF occurs. The 8-class model is chosen for its clinical relevance.

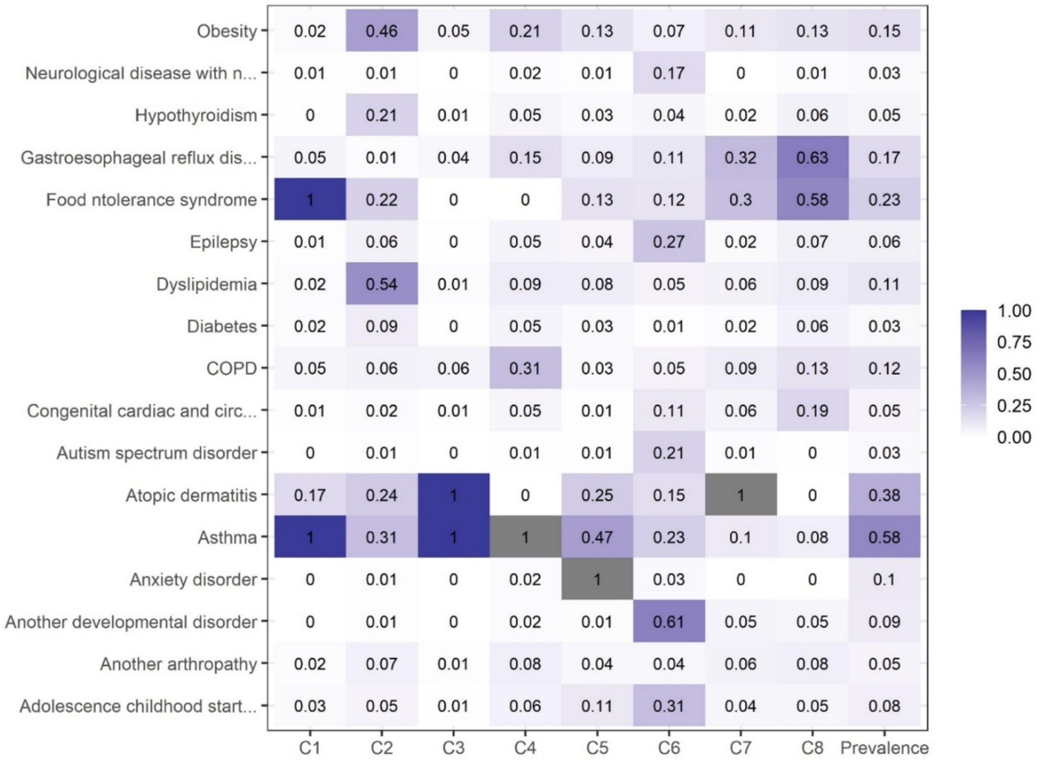

Supplementary Figure 3 | Likelihood of Belonging to each Multimorbidity Pattern of the Selected Chronic Conditions in the <16 and women model. The probability ranges from 0 to 1 and a stronger blue colour indicates a higher probability.

In view of the prevalence of the diseases in the Supplementary Figure 3, we can label each pattern as follows:

1) Asthma + Food Intolerance, 2) Cardiometabolic, 3) Asthma + Dermatitis, 4) Respiratory, 5) Mental, 6) Developmental + Neurological Problems, 7) Dermatitis + Digestive, 8) Digestive

2) <16 and men

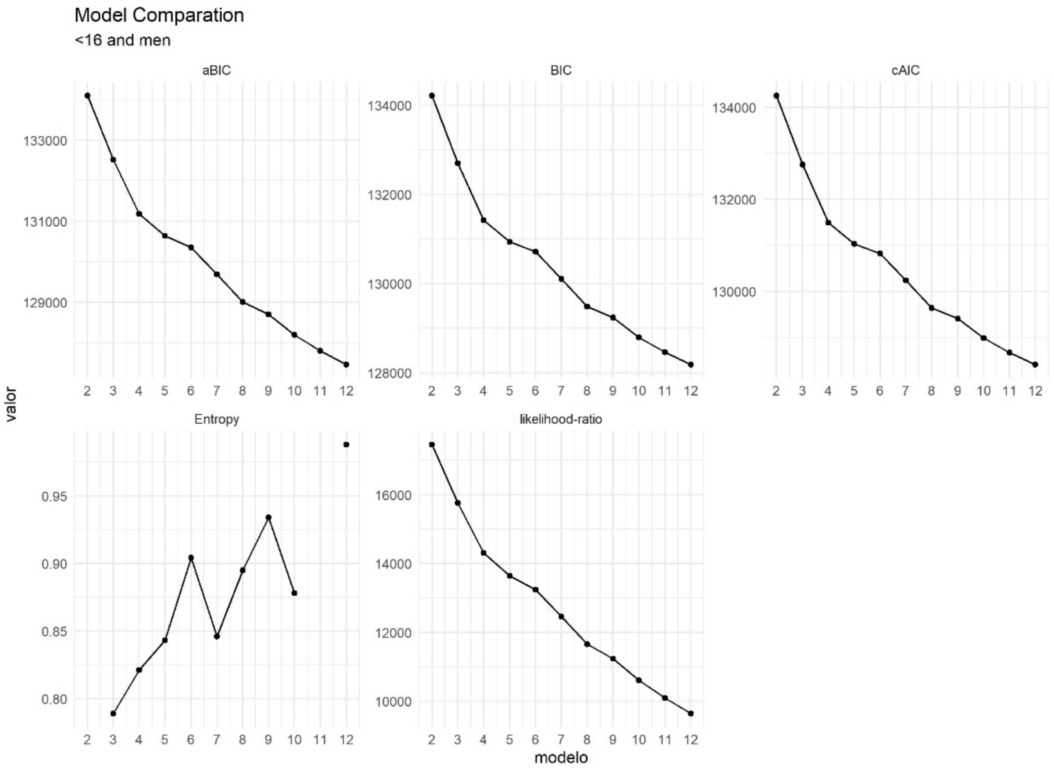

Supplementary Figure 4 | Godness-of-Fit Indices in the LCA Model with <16 and men. Looking at the lines, the chosen model is determined by trying to find the point where the slope starts to decrease the less

We can choose between models with more than 8 classes, which is where the decrease in GoF decrement occurs (Supplementary Figure 4). Models with 9 or more classes are not clinically relevant in this case, so the 8-class model is chosen.

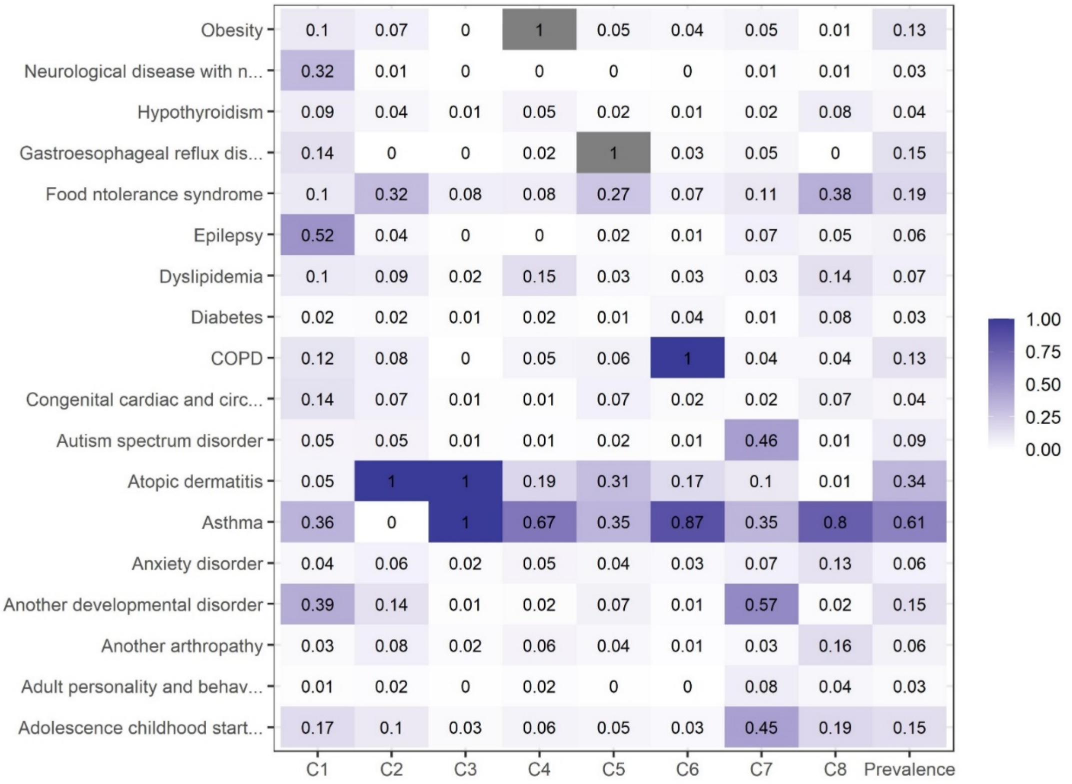

Supplementary Figure 5 | Likelihood of Belonging to each Multimorbidity Pattern of the Selected Chronic Conditions in the <16 and men model. The probability ranges from 0 to 1 and a stronger blue colour indicates a higher probability.

In view of the prevalence of the diseases in the Supplementary Figure 5, we can label each pattern as follows:

1) Developmental + Neurological Problems 2) Dermatitis + Food Intolerance 3) Asthma + Dermatitis, 4) Asthma + Obesity, 5) Digestive, 6) Respiratory 7) Mental + Developmental Problems, 8) Asthma + Food Intolerance

3) 16-44 and women

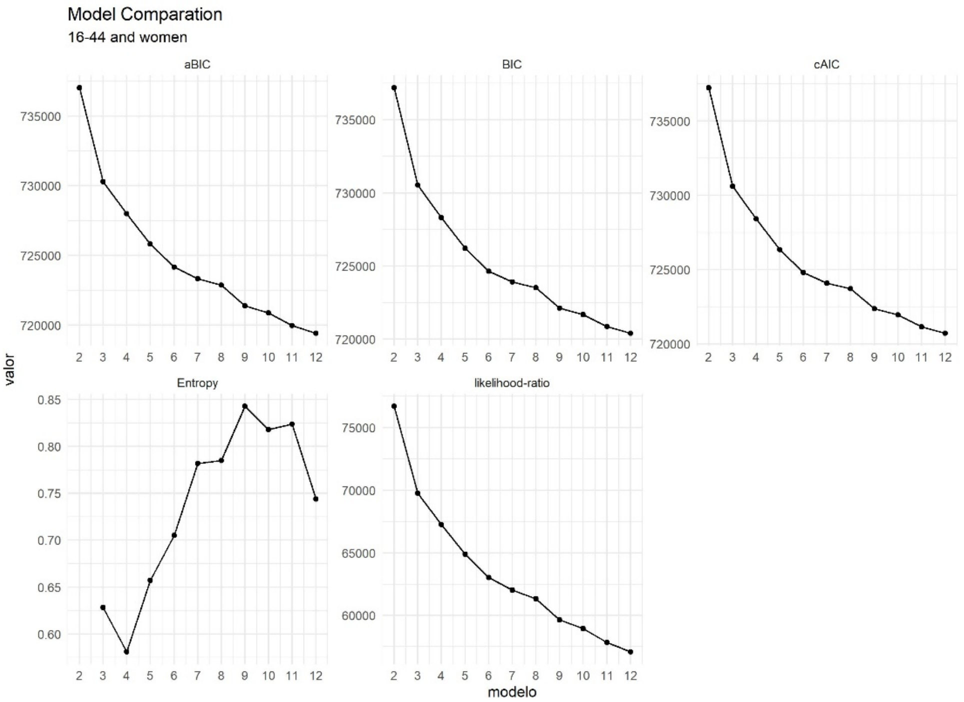

Supplementary Figure 6 | Godness-of-Fit Indices in the LCA Model with 16-44 and women. Looking at the lines, the chosen model is determined by trying to find the point where the slope starts to decrease the less

We can choose between the 7-, 8-, 9- and 10-class models, which is where the decrease in GoF decrement occurs (Supplementary Figure 6). Models with 8 or more classes do not provide clinical relevance in this case, so the 7-class model is chosen.

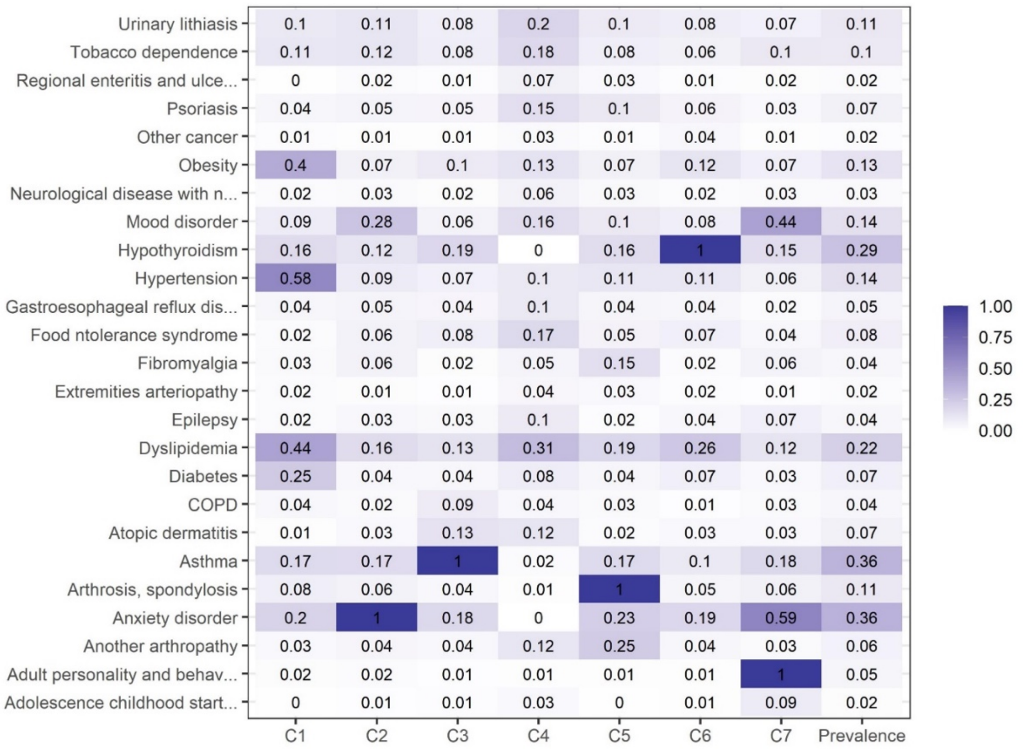

Supplementary Figure 7 | Likelihood of Belonging to each Multimorbidity Pattern of the Selected Chronic Conditions in the 16-44 and women model. The probability ranges from 0 to 1 and a stronger blue colour indicates a higher probability.

In view of the prevalence of the diseases in the Supplementary Figure 7, we can label each pattern as follows:

1) Cardiometabolic, 2) Mental, 3) Respiratory, 4) Digestive, 5) Musculoskeletal, 6) Dyslipidemia + Hypothyroidism, 7) Mental + Developmental Problems

4) 16-44 and men

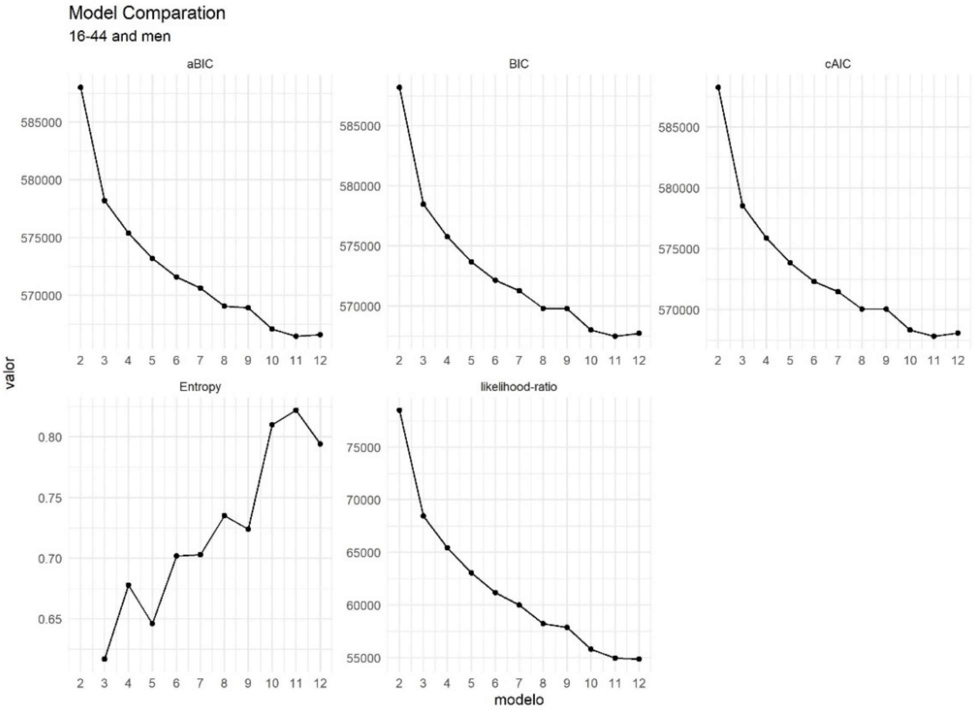

Supplementary Figure 8 | Godness-of-Fit Indices in the LCA Model with 16-44 and men. Looking at the lines, the chosen model is determined by trying to find the point where the slope starts to decrease the less

We can choose between the 8- and 9-class models, which is where the decrease in GoF decrement occurs (Supplementary Figure 8). The 8-class model is chosen because they are all clinically relevant.

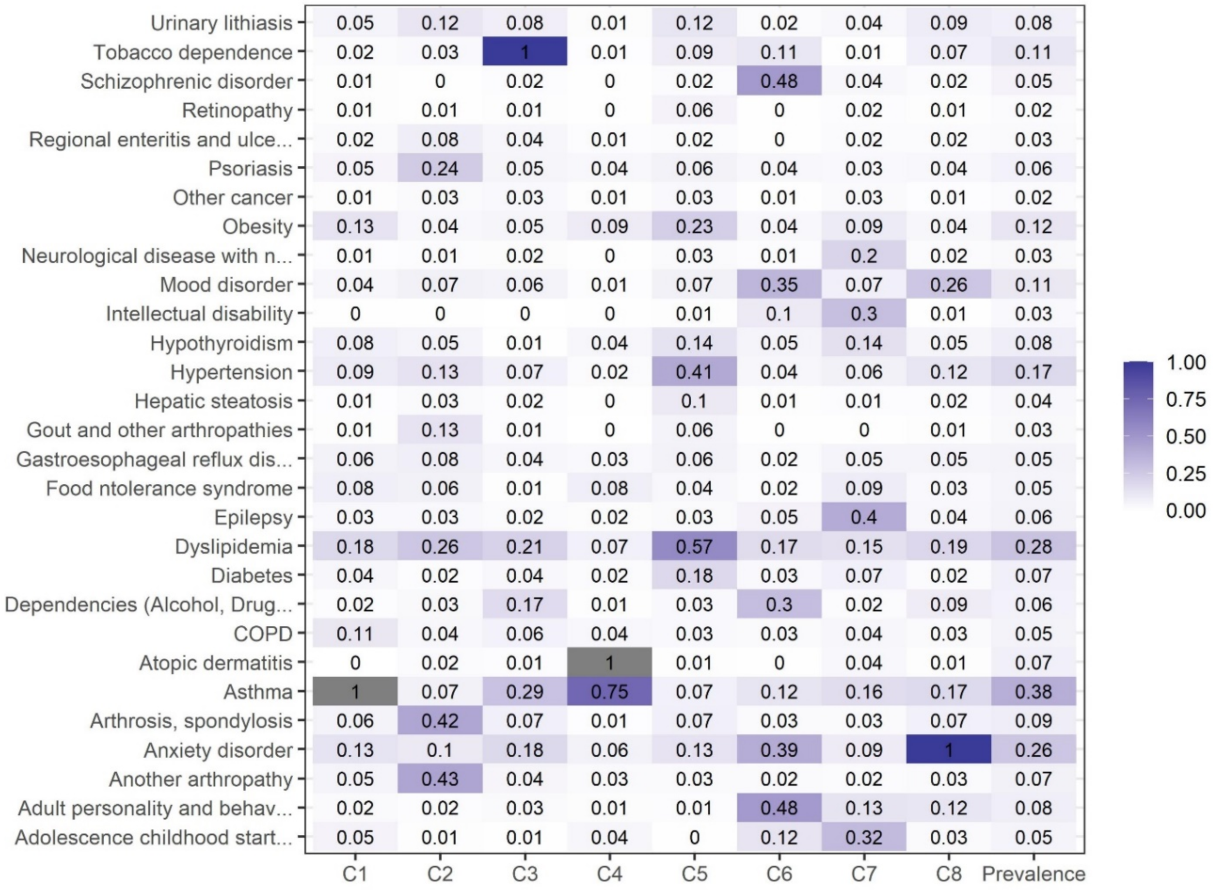

Supplementary Figure 9 | Likelihood of Belonging to each Multimorbidity Pattern of the Selected Chronic Conditions in the 16-44 and men model. The probability ranges from 0 to 1 and a stronger blue colour indicates a higher probability.

In view of the prevalence of the diseases in the supplementary Figure 9, we can label each pattern as follows:

1) Respiratory, 2) Musculoskeletal, 3) Asthma + Dependencies, 4) Asthma + Dermatitis, 5) Cardiometabolic, 6) Mental + Developmental Problems, 7) Developmental & Neurological Problems, 8) Mental

5) 45-64 and women

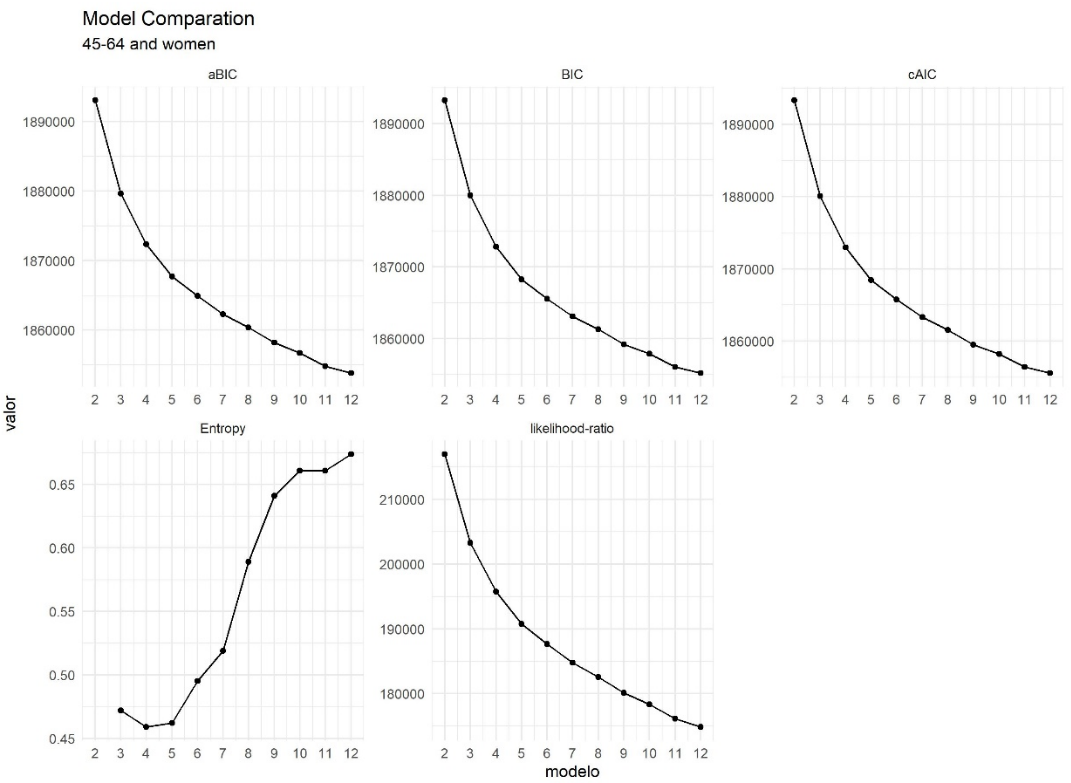

Supplementary Figure 10 | Godness-of-Fit Indices in the LCA Model with 45-64 and women. Looking at the lines, the chosen model is determined by trying to find the point where the slope starts to decrease the less

We can choose between models from 9 classes upwards, which is where the decrease in GoF decrement occurs (Supplementary Figure 10). Models with more than 9 classes are not clinically relevant, so the 9-class model is chosen.

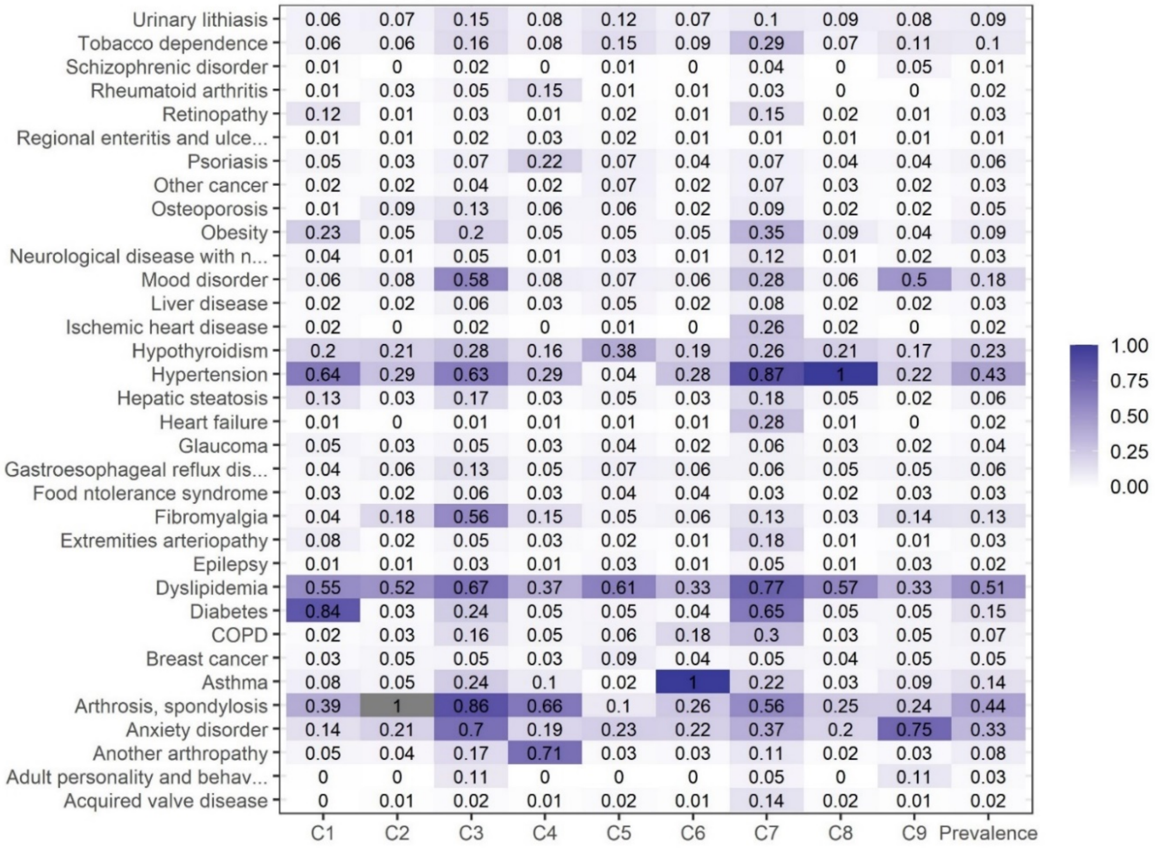

Supplementary Figure 11 | Likelihood of Belonging to each Multimorbidity Pattern of the Selected Chronic Conditions in the 45-64 and women model. The probability ranges from 0 to 1 and a stronger blue colour indicates a higher probability.

In view of the prevalence in the Supplementary Figure 11, we can label each pattern as follows:

1) Cardiometabolic + Retinopathy, 2) Hypertension + Dyslipidemia + Arthrosis, 3) Cardiometabolic + Mental + Musculoskeletal + Respiratory (Complex), 4) Musculoskeletal, 5) Dyslipidemia + Hypothyroidism, 6) Respiratory, 7) Cardiometabolic + Cardiovascular + Musculoskeletal + Mental (Complex), 8) Cardiometabolic, 9) Mental

6) 45-64 and men

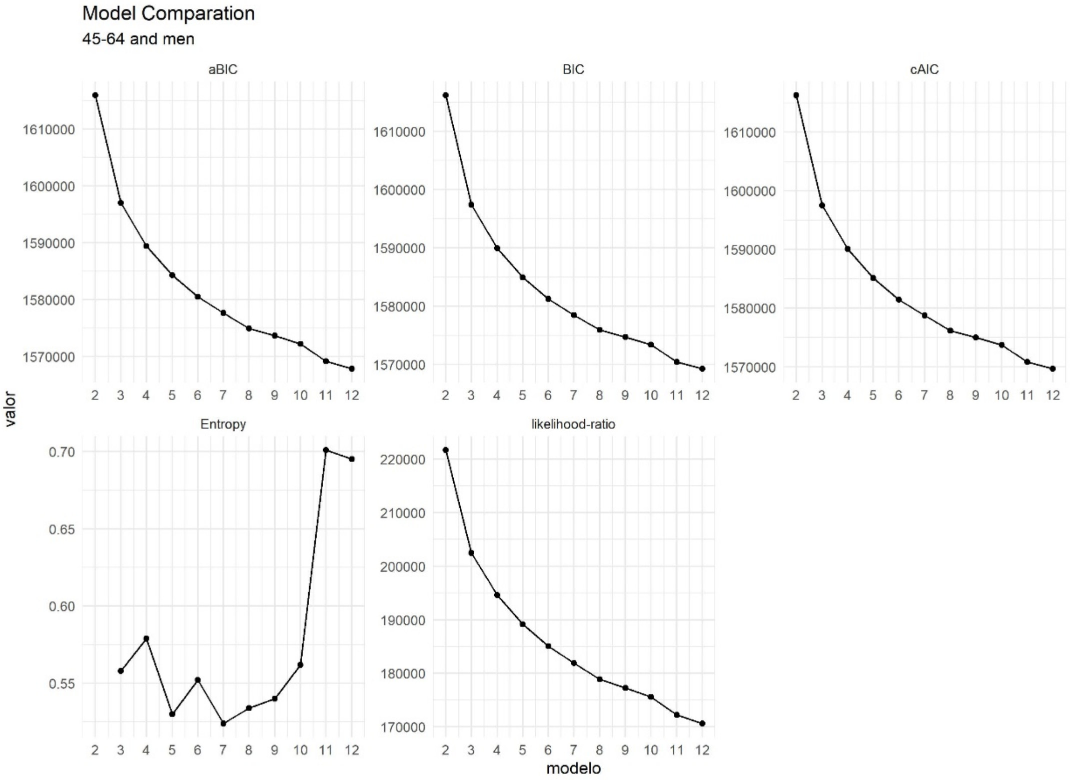

Supplementary Figure 12 | Godness-of-Fit Indices in the LCA Model with 45-64 and men. Looking at the lines, the chosen model is determined by trying to find the point where the slope starts to decrease the less

A choice can be made between models of 8 classes and upwards, which is where the decrease in GoF decrement occurs (Supplementary Figure 12). Models with 9 or more classes are not clinically relevant, so the 8-class model is chosen.

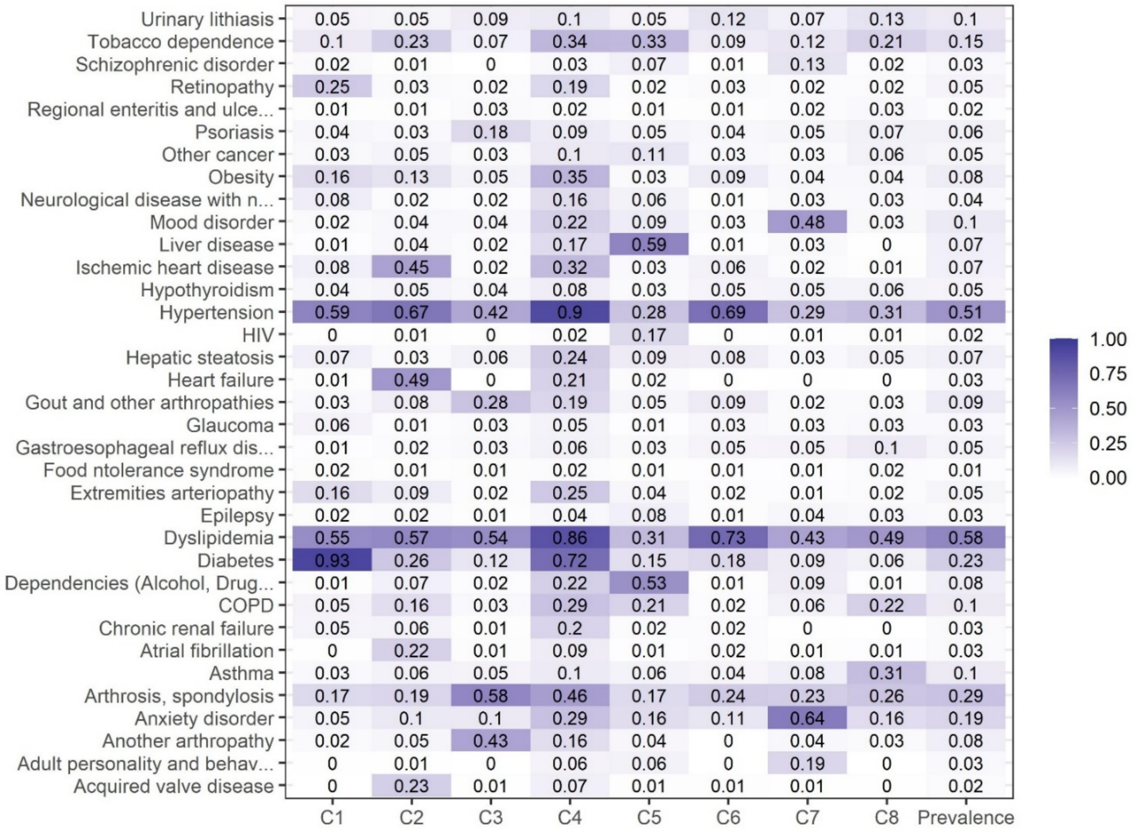

Supplementary Figure 13 | Likelihood of Belonging to each Multimorbidity Pattern of the Selected Chronic Conditions in the 45-64 and men model. The probability ranges from 0 to 1 and a stronger blue colour indicates a higher probability.

In view of the prevalence of the diseases in Supplementary Figure 13, we can label each pattern as follows:

1) Cardiometabolic + Retinopathy, 2) Cardiometabolic + Cardiovascular, 3) Musculoskeletal, 4) Cardiometabolic + Cardiovascular + Musculoskeletal + Mental (Complex), 5) Dependencies + Liver Disease, 6) Cardiometabolic, 7) Mental, 8) Respiratory

7) 65-79 and women

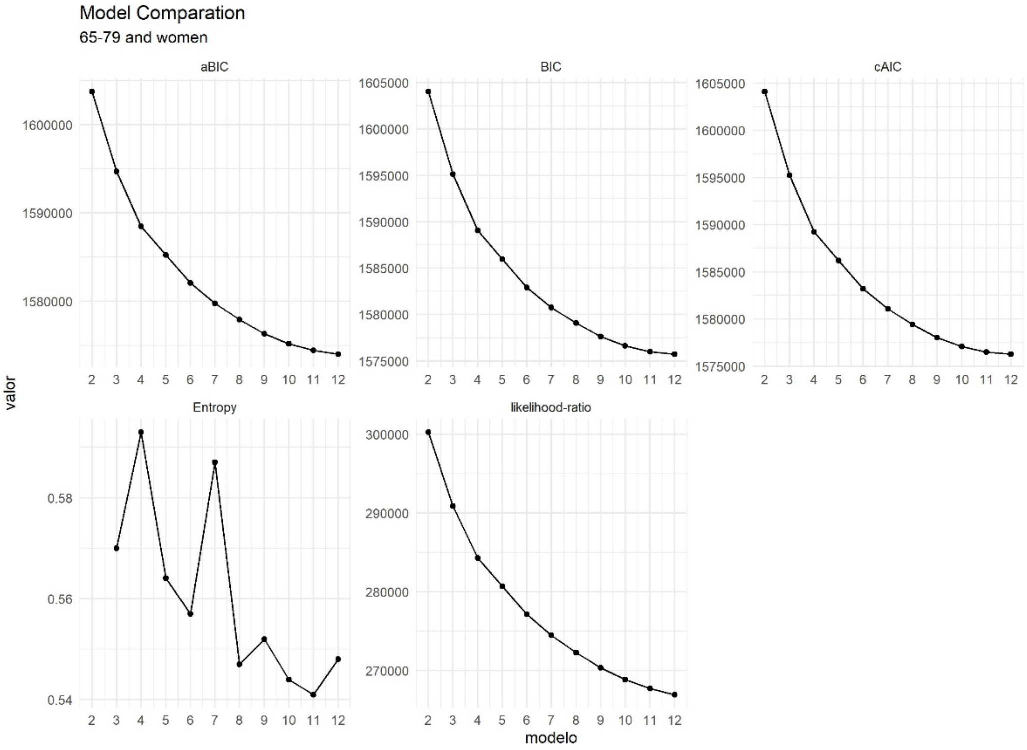

Supplementary Figure 14 | Godness-of-Fit Indices in the LCA Model with 65-79 and women. Looking at the lines, the chosen model is determined by trying to find the point where the slope starts to decrease the less

We can choose from 7-class models, which is where the decrease in GoF decrement occurs (Supplementary Figure 14). Models of 8 or more classes do not provide clinical relevance in this case, so the 7-class model is chosen.

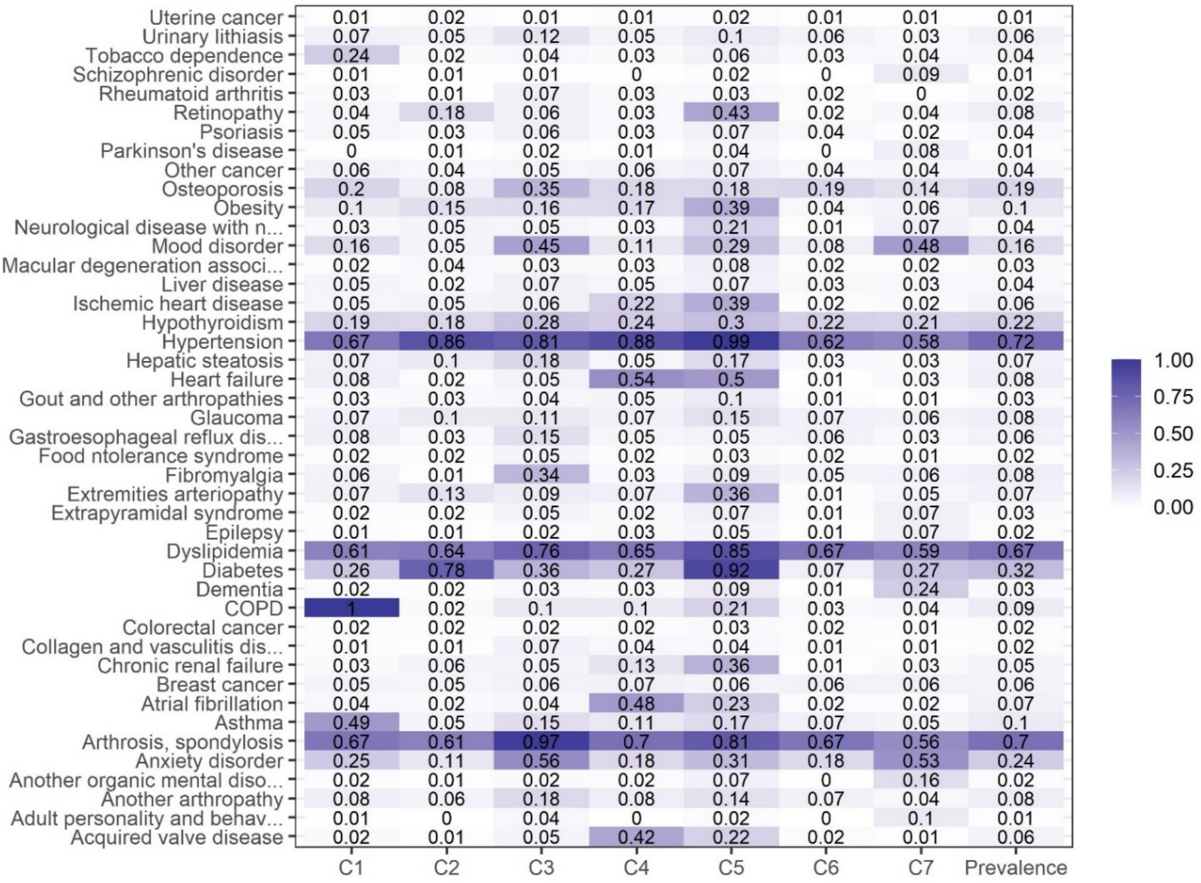

Supplementary Figure 15 | Likelihood of Belonging to each Multimorbidity Pattern of the Selected Chronic Conditions in the 65-79 and women model. The probability ranges from 0 to 1 and a stronger blue colour indicates a higher probability.

In view of the prevalence of the diseases in Supplementary Figure 15, we can label each pattern as follows:

1) Respiratory, 2) Cardiometabolic and Retinopathy, 3) Cardiometabolic + Mental + Musculoskeletal + Respiratory (Complex), 4) Cardiometabolic + Cardiovascular, 5) Cardiometabolic + Cardiovascular + Musculoskeletal + Mental (Complex), 6) Hypertension + Dyslipidemia + Arthrosis, 7) Mental

8) 65-79 and men

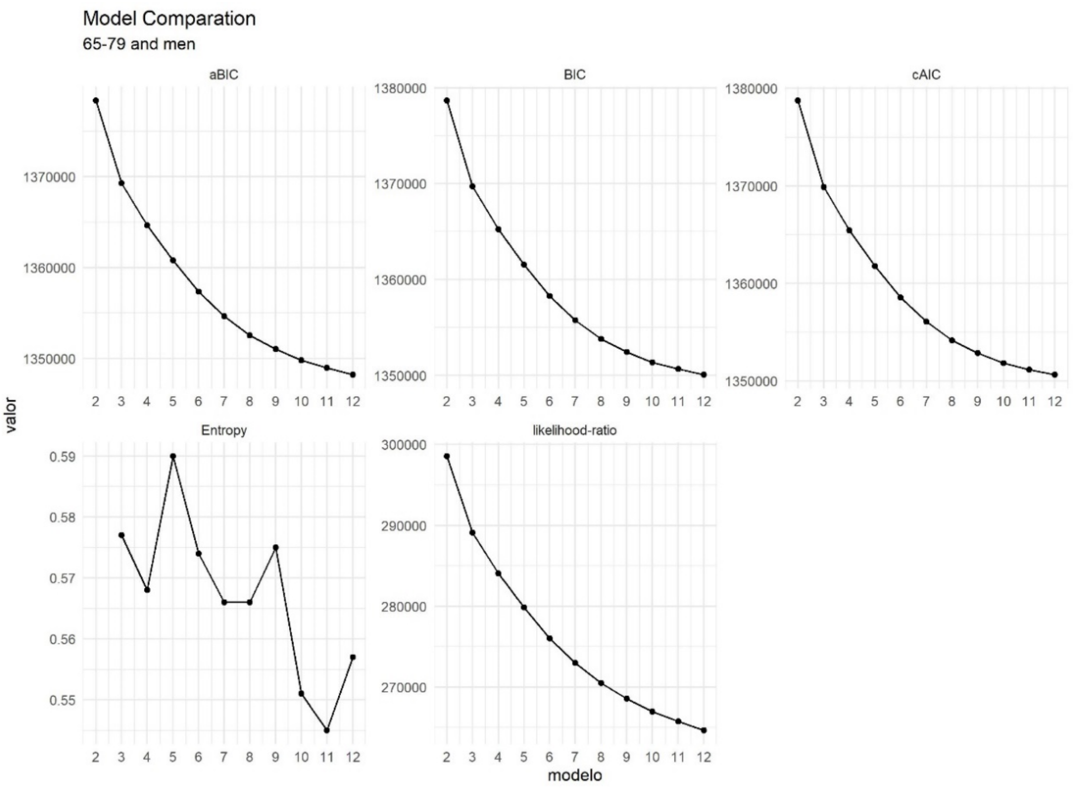

Supplementary Figure 16 | Godness-of-Fit Indices in the LCA Model with 65-79 and men. Looking at the lines, the chosen model is determined by trying to find the point where the slope starts to decrease the less

We can choose between models with 8 or more classes, which is where the decrease in GoF decrement occurs (Supplementary Figure 16). Models with 9 or more classes are not clinically relevant, so the 8-class model is chosen.

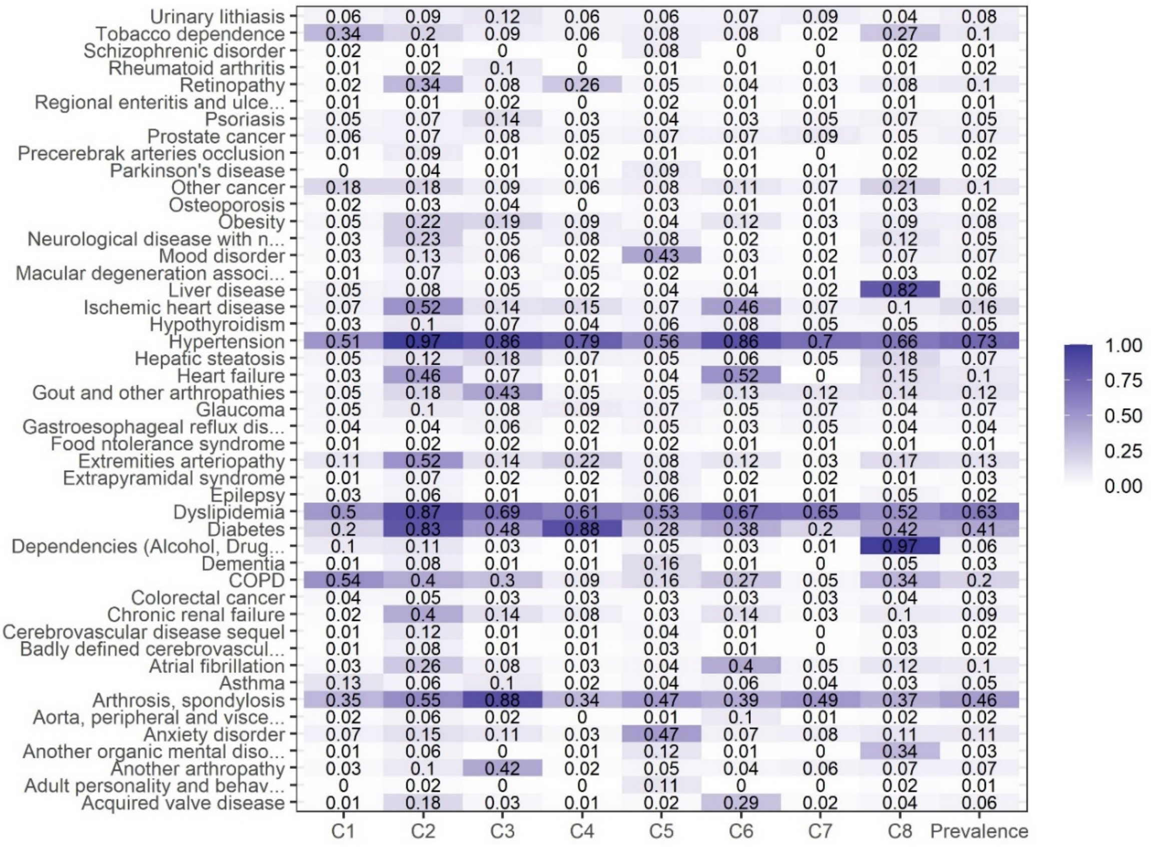

Supplementary Figure 17 | Likelihood of Belonging to each Multimorbidity Pattern of the Selected Chronic Conditions in the 65-79 and men model. The probability ranges from 0 to 1 and a stronger blue colour indicates a higher probability.

In view of the prevalence of the diseases in Supplementary Figure 17, we can label each pattern as follows:

1) Respiratory, 2) Cardiometabolic + Cardiovascular + Musculoskeletal + Respiratory (Complex), 3) Cardiometabolic + Musculoskeletal, 4) Cardiometabolic + Retinopathy, 5) Mental, 6) Cardiovascular, 7) Hypertension + Dyslipidemia + Arthrosis, 8) Dependencies + Liver disease

9) >79 and women

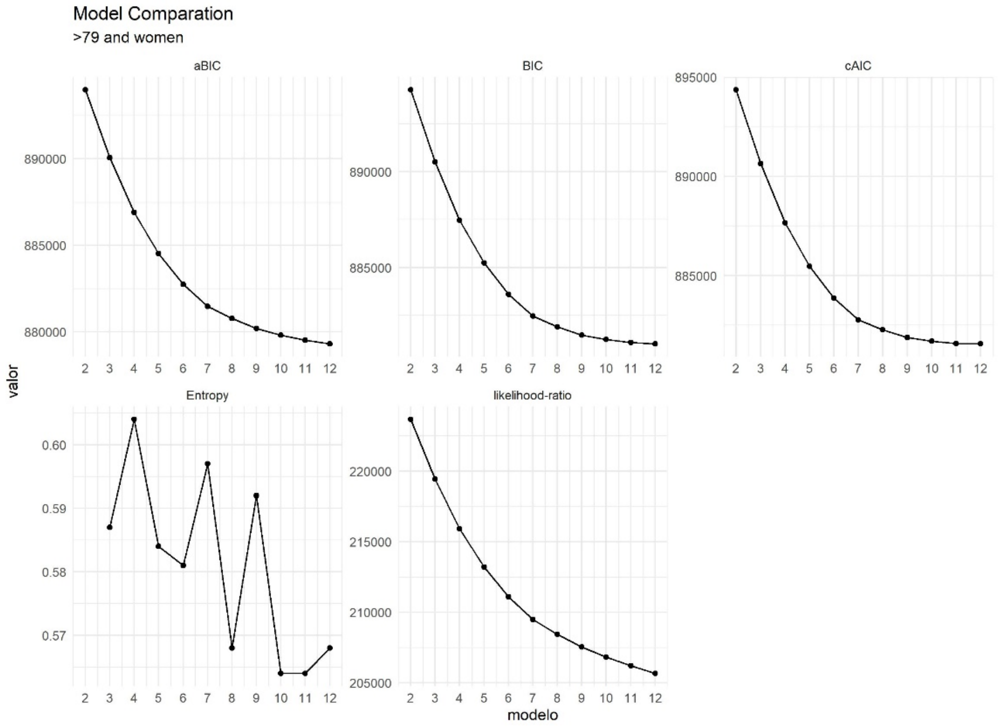

Supplementary Figure 18 | Godness-of-Fit Indices in the LCA Model with >79 and women. Looking at the lines, the chosen model is determined by trying to find the point where the slope starts to decrease the less

We can choose between models with more than 7 classes, which is where the decrease in GoF decrement occurs (Supplementary Figure 18). Models with 8 or more classes are not clinically relevant in this case, so the 7-class model is chosen.

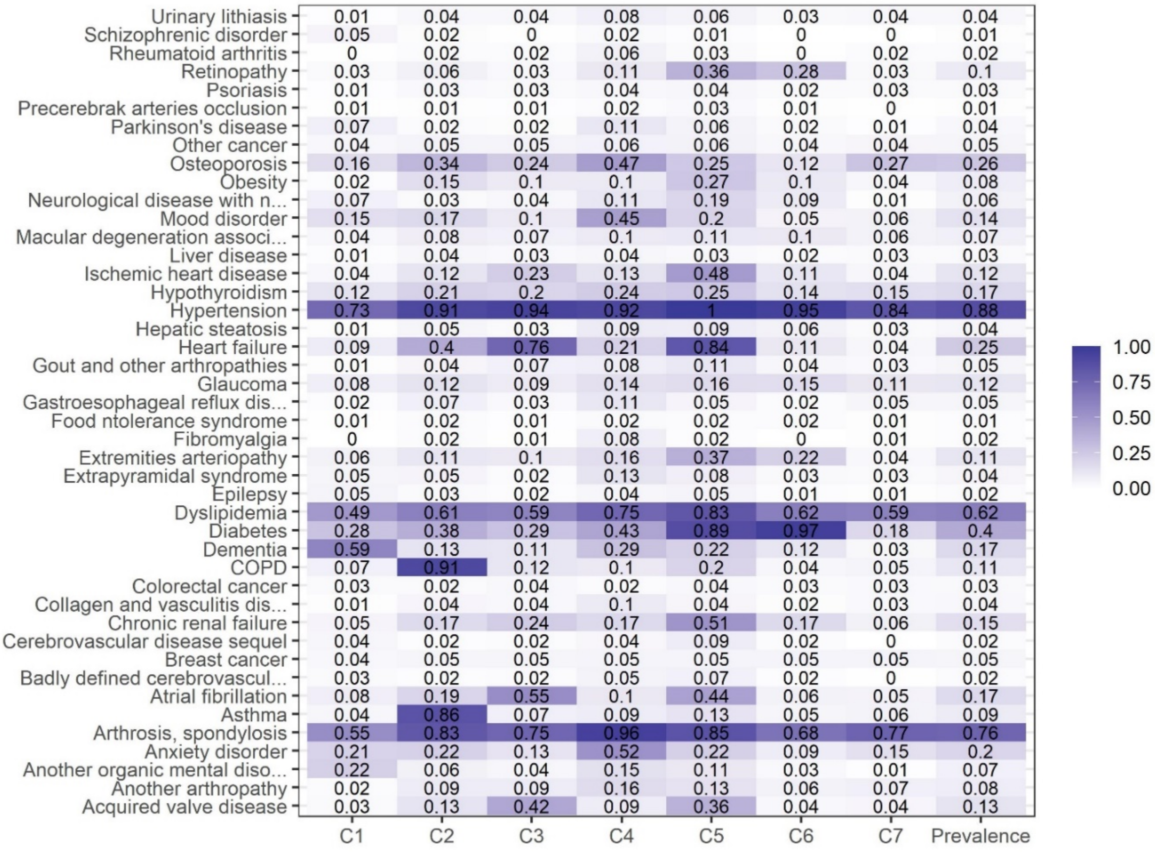

Supplementary Figure 19 | Likelihood of Belonging to each Multimorbidity Pattern of the Selected Chronic Conditions in the >79 and women model. The probability ranges from 0 to 1 and a stronger blue colour indicates a higher probability.

In view of the prevalence of the diseases in the Supplementary Figure 19, we can label each pattern as follows:

1) Dementia + Mental, 2) Cardiometabolic + Cardiovascular + Musculoskeletal + Respiratory (Complex), 3) Cardiovascular, 4) Cardiometabolic + Mental + Musculoskeletal + Respiratory (Complex), 5) Cardiometabolic + Cardiovascular + Musculoskeletal + Respiratory + Retinopathy + Renal (Complex) 6) Cardiometabolic + Retinopathy, 7) Hypertension + Dyslipidemia + Arthrosis

10) >79 and men

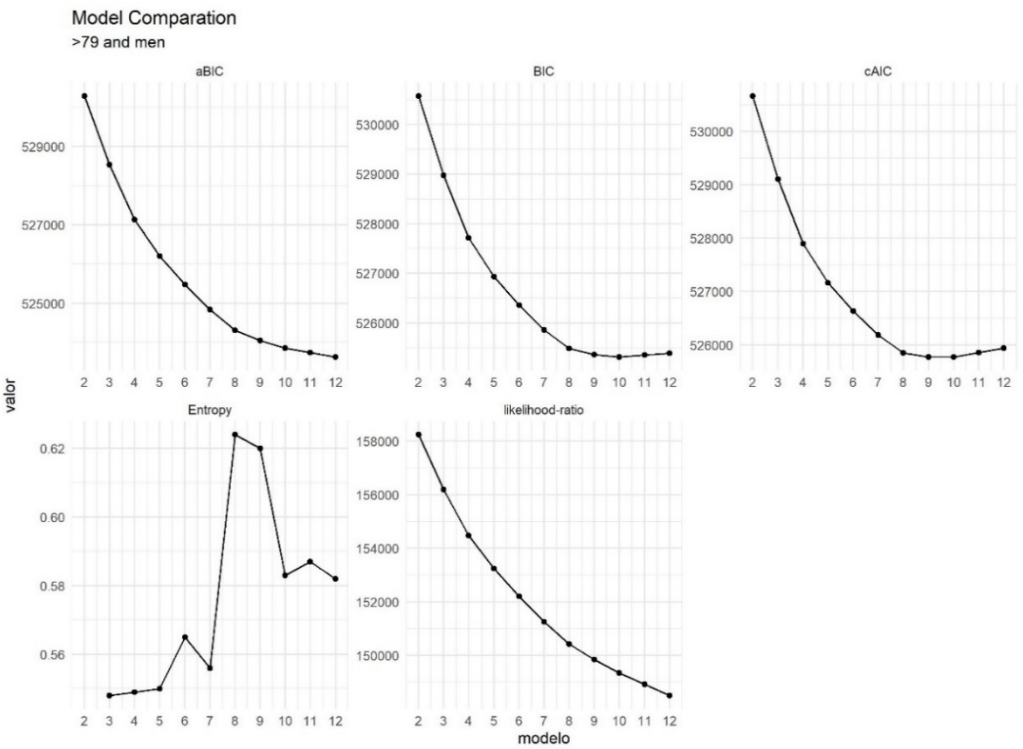

Supplementary Figure 20 | Godness-of-Fit Indices in the LCA Model with >79 and men. Looking at the lines, the chosen model is determined by trying to find the point where the slope starts to decrease the less

We can choose between models with more than 8 classes, which is where the decrease in GoF decrement occurs (Supplementary Figure 20). Models with 9 or more classes are not clinically relevant, so the 8-class model is chosen.

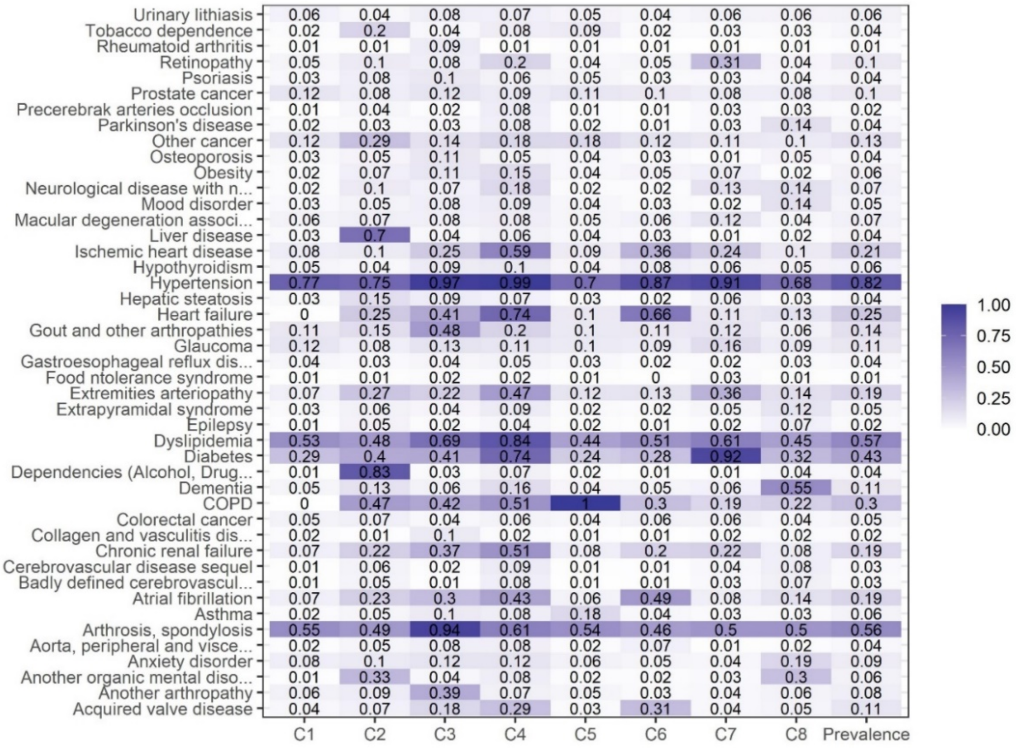

Supplementary Figure 21 | Likelihood of Belonging to each Multimorbidity Pattern of the Selected Chronic Conditions in the >79 and men model. The probability ranges from 0 to 1 and a stronger blue colour indicates a higher probability.

In view of the prevalence of the diseases, we can label each pattern as follows:

1) Hypertension + Dyslipidemia + Arthrosis 2) Dependencies + Liver Disease, 3) Cardiometabolic + Cardiovascular + Cardiometabolic + Cardiovascular + Musculoskeletal + Respiratory (Complex), 4) Cardiometabolic + Cardiovascular + Musculoskeletal + Respiratory + Retinopathy + Renal (Complex), 5) Respiratory, 6) Cardiovascular, 7) Cardiometabolic + Retinopathy, 8) Dementia + Mental

2. Supplementary Tables, *Characterisation of multimorbidity patterns by local health area in South Spain: epidemiology and impact on the use of health services.*

Supplementary Table 1. People distribution among multimorbidity patterns

Pattern	<16 W	16-44 W	45-64 W	65-79 W	>79 W	<16 M	16-44 M	45-64 M	65-79 M	>79 M
Asthma + Food Intolerance	643 (8.29)					2447 (21.49)				
Cardiometabolic	641 (8.27)	3760 (7.75)	18507 (18.6)				9313 (25.56)	34704 (41.34)		
Asthma + Dermatitis	1431 (18.45)					1925 (16.9)	2431 (6.67)			
Respiratory	1745 (22.5)	15061 (31.06)	9749 (9.8)	2776 (3.97)		1135 (9.97)	10171 (27.91)	12267 (14.61)	6898 (12.07)	2430 (12.65)
Mental	748 (9.65)	9834 (20.28)	14577 (14.65)	3208 (4.59)			5394 (14.8)	10572 (12.59)	3507 (6.14)	
Developmental Problems + Neurological	808 (10.42)					498 (4.37)	1748 (4.8)			
Dermatitis + Digestive	1010 (13.03)									
Digestive	728 (9.39)	6148 (12.68)				1498 (13.15)				
Dermatitis + Food Intolerance						941 (8.26)				
Asthma + Obesity						1233 (10.83)				
Mental + Developmental Problems		1955 (4.03)				1712 (15.03)	2535 (6.96)			
Hypothyroidism + Dyslipidemia		8825 (18.2)	13456 (13.52)							
Musculoskeletal		2907 (6)	5410 (5.44)				2039 (5.6)	7019 (8.36)		
Asthma + Dependence							2807 (7.7)			
Hypertension + Dyslipidemia + Arthrosis			20020 (20.12)	36123 (51.64)	14005 (40.57)				23620 (41.33)	6914 (35.99)
Cardiometabolic + Rethinopathy			9687 (9.73)	13310 (19.03)	4828 (13.99)			6251 (7.45)	9666 (16.91)	2379 (12.38)
Cardiometabolic + Mental + Musculoskeletal + Respiratory (Complex)			5572 (5.6)	7433 (10.63)	3600 (10.43)					
Cardiometabolic + Cardiovascular + Musculoskeletal + Mental (Complex)			2532 (2.54)	3179 (4.54)				3763 (4.48)		
Cardiometabolic + Cardiovascular				3922 (5.61)				2352 (2.8)		
Dependence + Liver Disease								7027 (8.37)	1501 (2.63)	397 (2.07)
Cardiovascular					4851 (14.05)				4781 (8.36)	2386 (12.42)
Cardiometabolic + Cardiovascular + Musculoskeletal + Respiratory (Complex)					1154 (3.34)				4174 (7.3)	1144 (5.95)
Cardiometabolic + Musculoskeletal									3009 (5.26)	
Dementia + Mental					3780 (10.95)					1459 (7.59)
Cardiometabolic + Cardiovascular + Musculoskeletal + Respiratory + Retinopathy + Renal (Complex)					2300 (6.66)					2104 (10.95)

Supplementary Table 2. Logistic regression models by SES area

Pattern	OR[1]	95% CI[1]	z	p-value	OR[1]	95% CI[1]	z	p-value	OR[1]	95% CI[1]	z	p-value
		**Low SES**				**Medium SES**				**High SES**		
Cardiometabolic (Ref.)	-	-	-		-	-	-		-	-	-	
Respiratory	1.54	0.90, 2.73	1.53	0.13	1.62	1.30, 2.05	4.16	<0.001***	1.37	1.05, 1.81	2.28	0.022*
Mental	2.41	1.38, 4.34	3.03	0.002**	2.3	1.82, 2.92	6.94	<0.001***	1.77	1.34, 2.35	3.95	<0.001***
Hypothyroidism + Dyslipidemia	3.22	1.37, 7.03	2.84	0.004**	2.67	1.87, 3.74	5.58	<0.001***	1.86	1.18, 2.84	2.77	0.006**
Musculoskeletal	0.92	0.27, 2.44	-0.16	0.87	1.05	0.67, 1.59	0.24	0.81	0.77	0.41, 1.34	-0.86	0.39
Hypertension + Dyslipidemia + Arthrosis	1.02	0.63, 1.76	0.09	>0.9	0.74	0.60, 0.93	-2.66	0.008**	0.58	0.45, 0.75	-4.17	<0.001***
Cardiometabolic + Rethinopathy	1.3	0.78, 2.26	0.96	0.34	1.24	0.99, 1.55	1.88	0.06	1.01	0.78, 1.32	0.05	>0.9
Cardiometabolic + Mental + Musculoskeletal + Respiratory (Complex)	1.58	0.89, 2.91	1.52	0.13	1.95	1.53, 2.51	5.33	<0.001***	1.31	0.97, 1.77	1.77	0.077·
Cardiometabolic + Cardiovascular + Musculoskeletal + Mental (Complex)	5.22	2.94, 9.51	5.56	<0.001***	5.79	4.57, 7.38	14.38	<0.001***	4.60	3.45, 6.18	10.27	<0.001***
Cardiometabolic + Cardiovascular	3.88	2.03, 7.44	4.12	<0.001***	2.9	2.18, 3.87	7.29	<0.001***	1.75	1.21, 2.52	3.01	0.003**
Dependence + Liver Disease	5.2	2.97, 9.40	5.63	<0.001***	6.36	5.03, 8.09	15.33	<0.001***	5.56	4.18, 7.45	11.63	<0.001***
Cardiovascular	2.72	1.61, 4.80	3.62	<0.001***	2.51	2.00, 3.16	7.87	<0.001***	1.71	1.30, 2.27	3.81	<0.001***
Cardiometabolic + Cardiovascular + Musculoskeletal + Respiratory (Complex)	2.2	1.24, 4.02	2.63	0.009**	2.96	2.33, 3.78	8.83	<0.001***	2.07	1.56, 2.78	4.94	<0.001***
Cardiometabolic + Musculoskeletal	0.55	0.13, 1.65	-0.95	0.342	1.13	0.75, 1.67	0.61	0.54	1.11	0.69, 1.74	0.45	0.65
Dementia + Mental	3.85	2.22, 6.94	4.66	<0.001***	3.38	2.66, 4.33	9.87	<0.001***	2.56	1.93, 3.44	6.38	<0.001***
Cardiometabolic + Cardiovascular + Musculoskeletal + Respiratory + Retinopathy + Renal (Complex)	3.57	2.05, 6.47	4.37	<0.001***	4.4	3.47, 5.61	12.15	<0.001***	2.94	2.22, 3.94	7.35	<0.001***

[1]OR = Odds Ratio, CI = Confidence Interval. ***, **, *, and · indicate significance level at 0.1%, 1%, 5%, and 10%, respectively, Sample Sizes are Low SES: 46939, Medium SES: 256133, High SES: 187058

3. Supplementary Maps, *Characterisation of multimorbidity patterns by local health area in South Spain: epidemiology and impact on the use of health services.*

We show the rest of the maps of the prevalence of the patterns obtained according to the local health area of the province of Cadiz. The patterns we show are the following:

P1: Asthma + Food Intolerance, P2: Cardiometabolic, P3: Asthma + Dermatitis, P6: Developmental Problems + Neurological, P7: Dermatitis + Digestive, P8: Digestive, P9: Dermatitis + Food Intolerance, P10: Asthma + Obesity, P11: Mental + Developmental Problems, P12: Hypothyroidism + Dyslipidemia, P13: Musculoskeletal, P14: Asthma + Dependencies, P17: Cardiometabolic + Mental + Musculoskeletal + Respiratory (Complex), P18: Cardiometabolic + Cardiovascular + Musculoskeletal + Mental (Complex), P19: Cardiometabolic + Cardiovascular , P21: Cardiovascular, P22: Cardiometabolic + Cardiovascular + Musculoskeletal + Respiratory (Complex), P23: Cardiometabolic + Musculoskeletal, P24: Dementia + Mental, P25: Cardiometabolic + Cardiovascular + Musculoskeletal + Respiratory + Retinopathy + Renal (Complex)

P1: Asthma + Food Intolerance

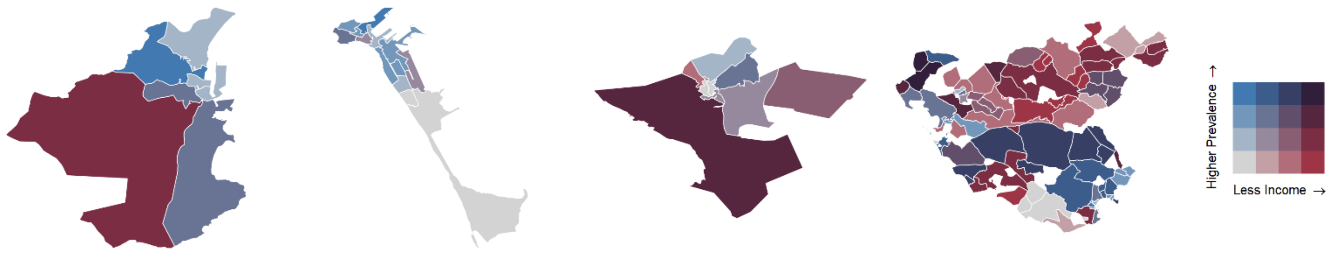

Supplementary Figure 22 | Prevalence of P1: Asthma + Food Intolerance by local health area. In the maps, we can see the absolute prevalence of the pattern within all persons with multimorbidity at the level of the whole province (right side map) and the level of the three main urban centres of the province (left side of the map).

P2: Cardiometabolic

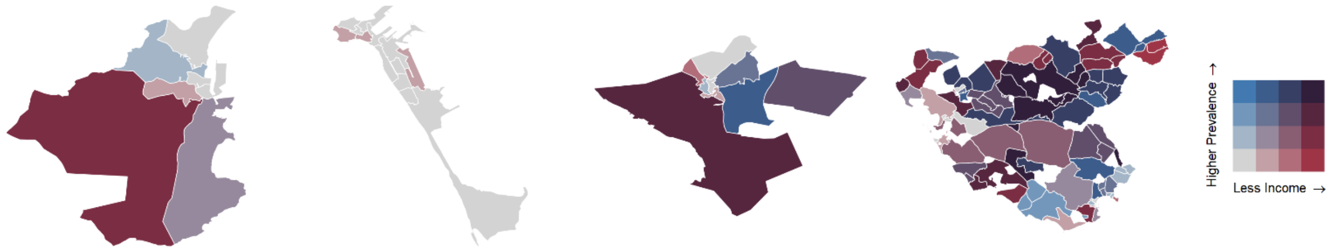

Supplementary Figure 23 | Prevalence of P2: Cardiometabolic by local health area. In the maps, we can see the absolute prevalence of the pattern within all persons with multimorbidity at the level of the whole province (right side map) and the level of the three main urban centres of the province (left side of the map).

P3: Asthma + Dermatitis

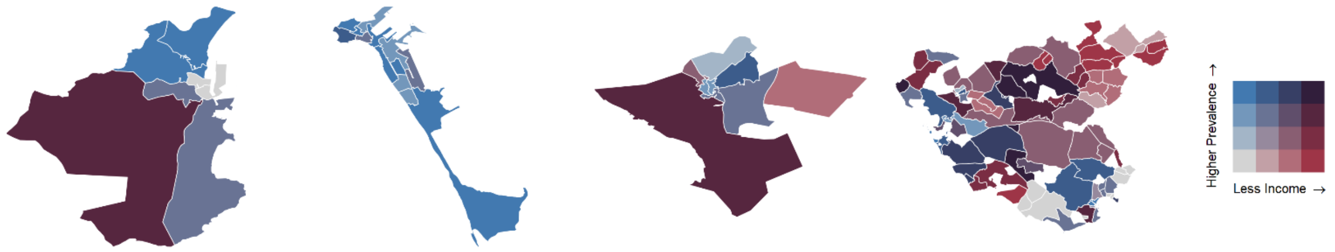

Supplementary Figure 24 | Prevalence of P3: Asthma + Dermatitis by local health area. In the maps, we can see the absolute prevalence of the pattern within all persons with multimorbidity at the level of the whole province (right side map) and the level of the three main urban centres of the province (left side of the map).

P4: Respiratory

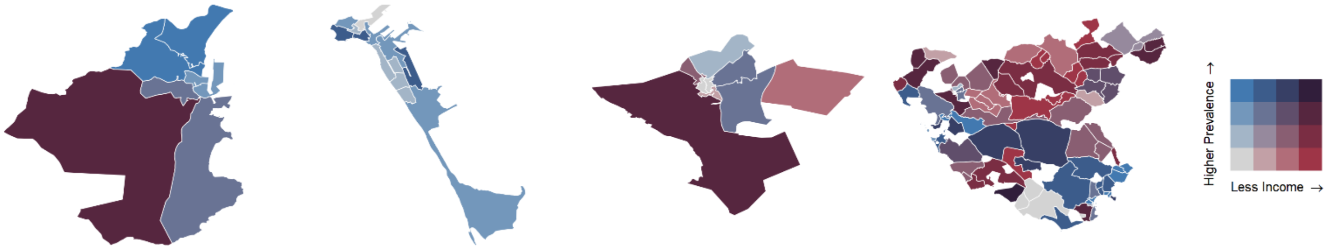

Supplementary Figure 25 | Prevalence of P4: Respiratory by local health area. In the maps, we can see the absolute prevalence of the pattern within all persons with multimorbidity at the level of the whole province (right side map) and the level of the three main urban centres of the province (left side of the map).

P5: Mental

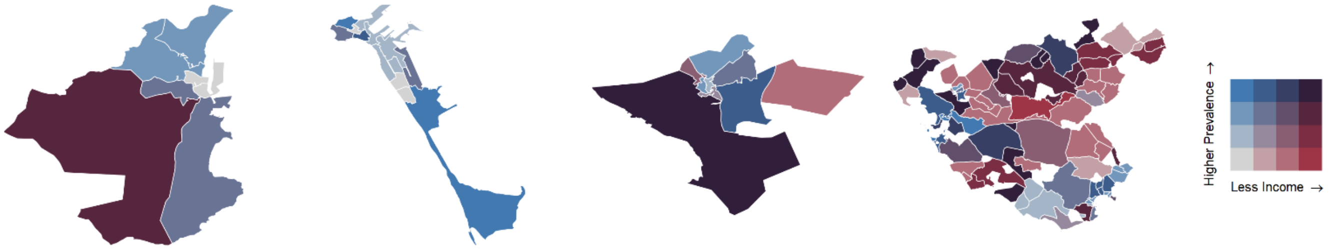

Supplementary Figure 26 | Prevalence of P5: Mental by local health area. In the maps, we can see the absolute prevalence of the pattern within all persons with multimorbidity at the level of the whole province (right side map) and the level of the three main urban centres of the province (left side of the map).

P6: Developmental Problems + Neurological

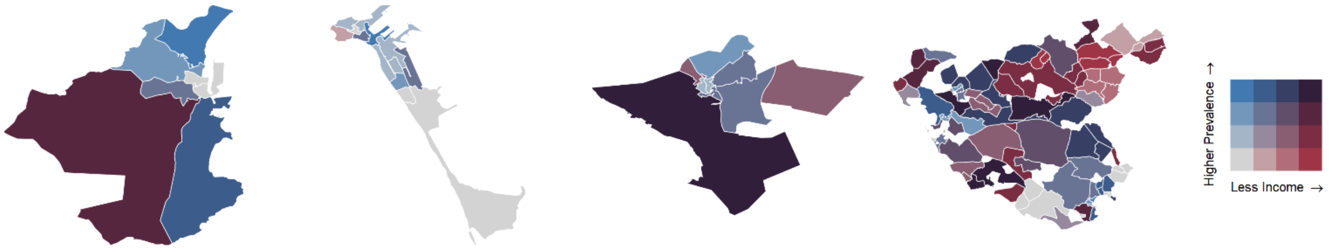

Supplementary Figure 27 | Prevalence of P6: Developmental Problems + Neurological by local health area. In the maps, we can see the absolute prevalence of the pattern within all persons with multimorbidity at the level of the whole province (right side map) and the level of the three main urban centres of the province (left side of the map).

P7: Dermatitis + Digestive

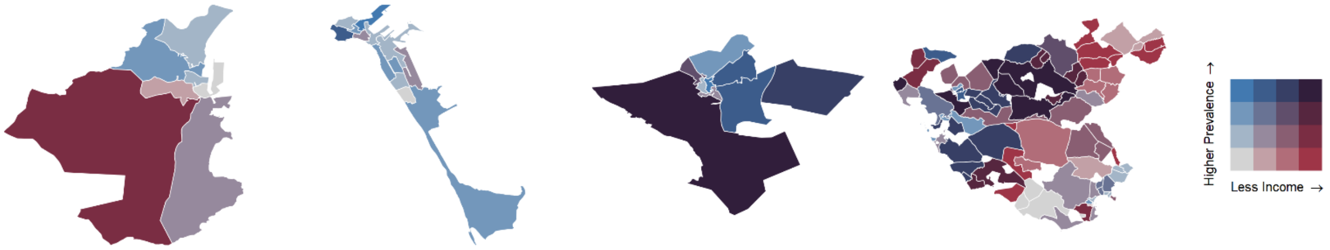

Supplementary Figure 28 | Prevalence of P7: Dermatitis + Digestive by local health area. In the maps, we can see the absolute prevalence of the pattern within all persons with multimorbidity at the level of the whole province (right side map) and the level of the three main urban centres of the province (left side of the map).

P8: Digestive

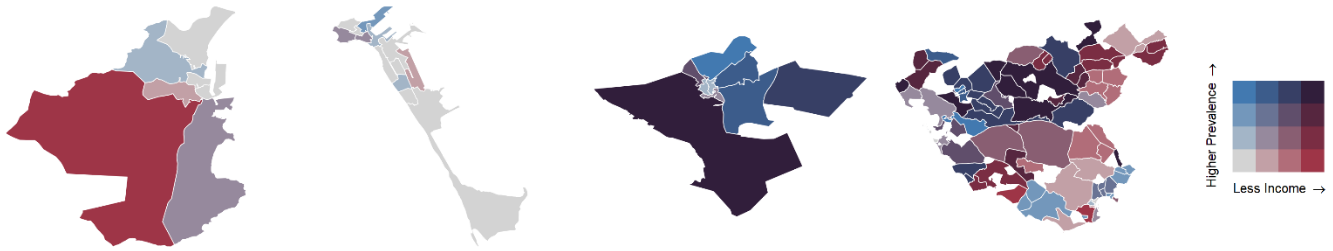

Supplementary Figure 29 | Prevalence of P8: Digestive by local health area. In the maps, we can see the absolute prevalence of the pattern within all persons with multimorbidity at the level of the whole province (right side map) and the level of the three main urban centres of the province (left side of the map).

P9: Dermatitis + Food Intolerance

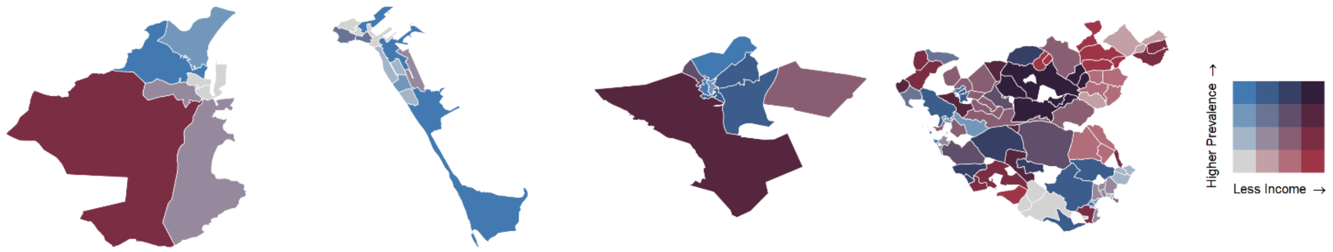

Supplementary Figure 30 | Prevalence of P9: Dermatitis + Food Intolerance by local health area. In the maps, we can see the absolute prevalence of the pattern within all persons with multimorbidity at the level of the whole province (right side map) and the level of the three main urban centres of the province (left side of the map).

P10: Asthma + Obesity

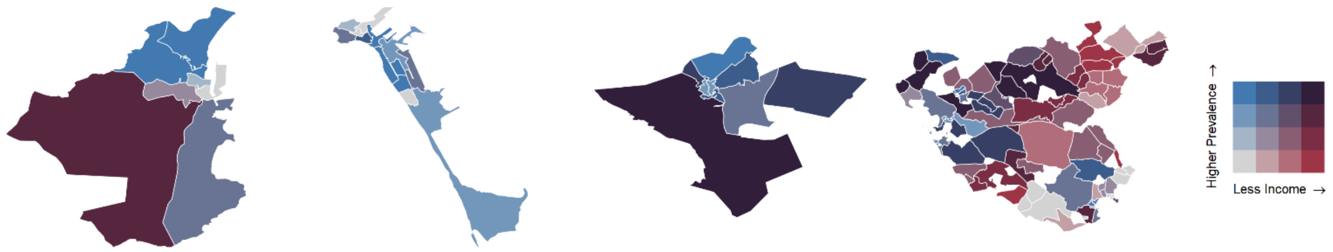

Supplementary Figure 31 | Prevalence of P10: Asthma + Obesity by local health area. In the maps, we can see the absolute prevalence of the pattern within all persons with multimorbidity at the level of the whole province (right side map) and the level of the three main urban centres of the province (left side of the map).

P11: Mental + Developmental Problems

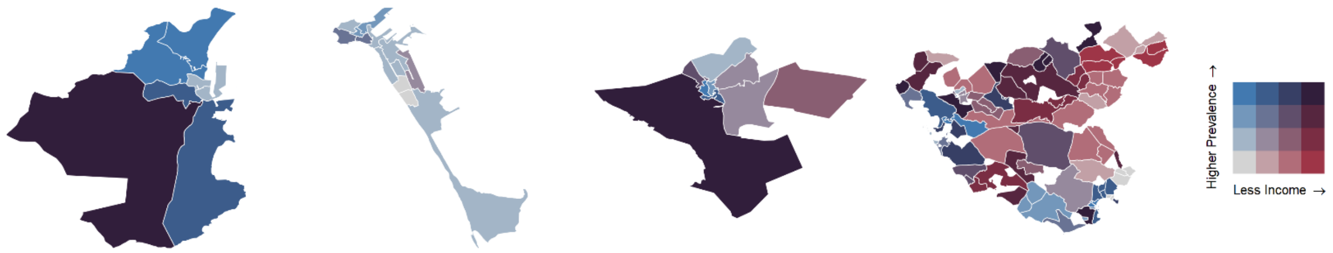

Supplementary Figure 32 | Prevalence of P11: Mental + Developmental Problems by local health area. In the maps, we can see the absolute prevalence of the pattern within all persons with multimorbidity at the level of the whole province (right side map) and the level of the three main urban centres of the province (left side of the map).

P12: Hypthyroidism + Dyslipidemia

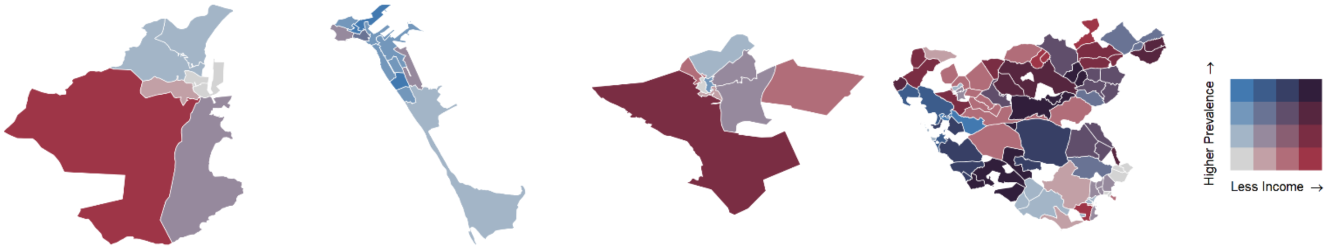

Supplementary Figure 33 | Prevalence of P12: Hypothyroidism + Dyslipidemia by local health area. In the maps, we can see the absolute prevalence of the pattern within all persons with multimorbidity at the level of the whole province (right side map) and the level of the three main urban centres of the province (left side of the map).

P13: Musculoskeletal

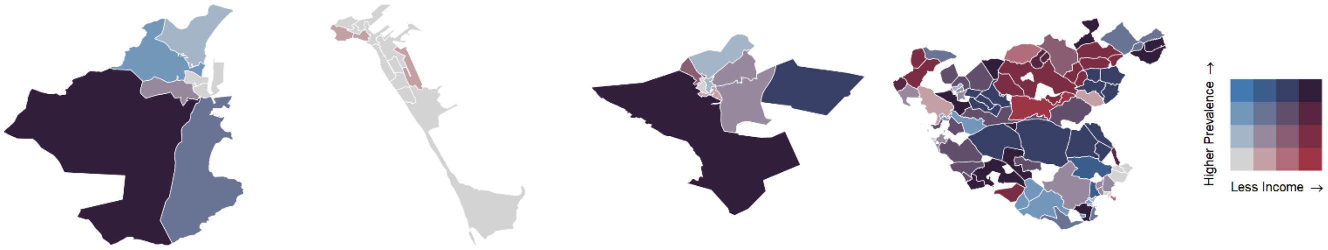

Supplementary Figure 34 | Prevalence of P13: Musculoskeletal by local health area. In the maps, we can see the absolute prevalence of the pattern within all persons with multimorbidity at the level of the whole province (right side map) and the level of the three main urban centres of the province (left side of the map).

P14: Asthma + Dependence

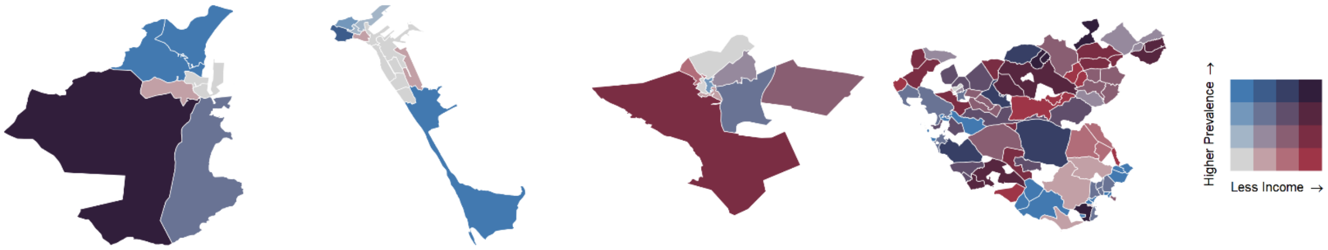

Supplementary Figure 35 | Prevalence of P14: Asthma + Dependence by local health area. In the maps, we can see the absolute prevalence of the pattern within all persons with multimorbidity at the level of the whole province (right side map) and the level of the three main urban centres of the province (left side of the map).

P15: Hypertension + Dyslipidemia + Arthrosis

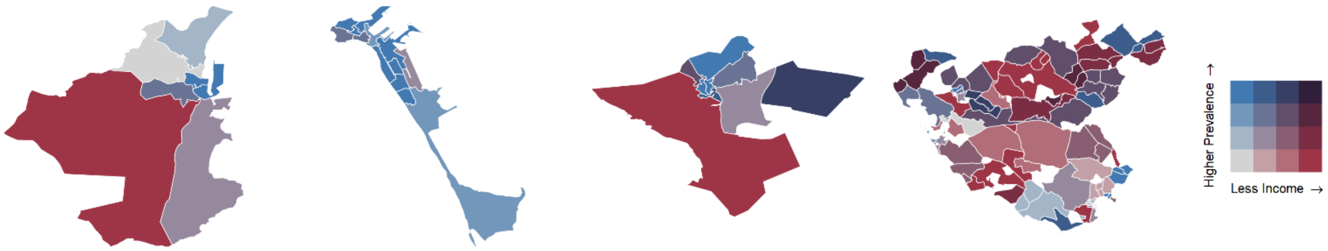

Supplementary Figure 36 | Prevalence of P15: Hypertension + Dyslipidemia + Arthrosis by local health area. In the maps, we can see the absolute prevalence of the pattern within all persons with multimorbidity at the level of the whole province (right side map) and the level of the three main urban centres of the province (left side of the map).

P16: Cardiometabolic + Rethinopathy

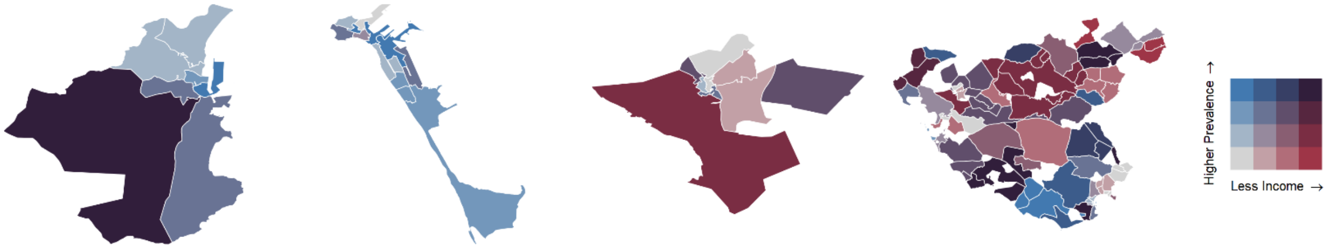

Supplementary Figure 37 | Prevalence of P16: Cardiometabolic + Rethinopathy by local health area. In the maps, we can see the absolute prevalence of the pattern within all persons with multimorbidity at the level of the whole province (right side map) and the level of the three main urban centres of the province (left side of the map).

P17: Cardiometabolic + Mental + Musculoskeletal + Respiratory (Complex)

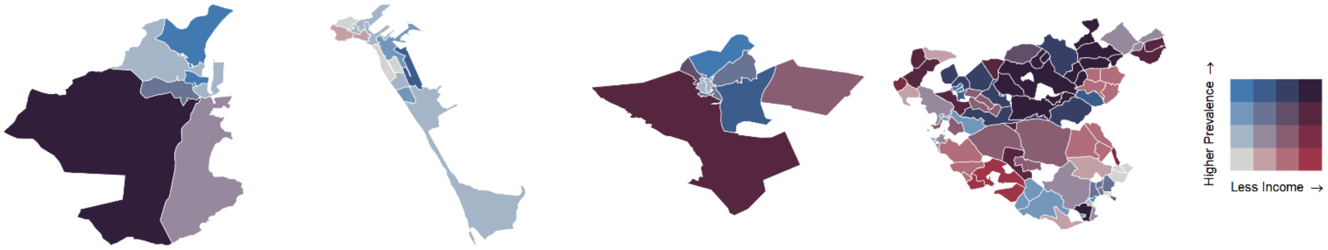

Supplementary Figure 38 | Prevalence of P17: Cardiometabolic + Mental + Musculoskeletal + Respiratory (Complex) by local health area. In the maps, we can see the absolute prevalence of the pattern within all persons with multimorbidity at the level of the whole province (right side map) and the level of the three main urban centres of the province (left side of the map).

P18: Cardiometabolic + Cardiovascular + Musculoskeletal + Mental (Complex)

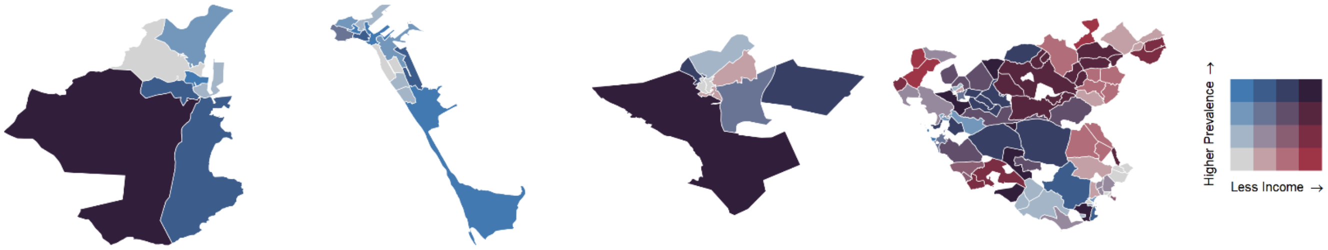

Supplementary Figure 39 | Prevalence of P18: Cardiometabolic + Cardiovascular + Musculoskeletal + Mental (Complex) by local health area. In the maps, we can see the absolute prevalence of the pattern within all persons with multimorbidity at the level of the whole province (right side map) and the level of the three main urban centres of the province (left side of the map).

P19: Cardiometabolic + Cardiovascular

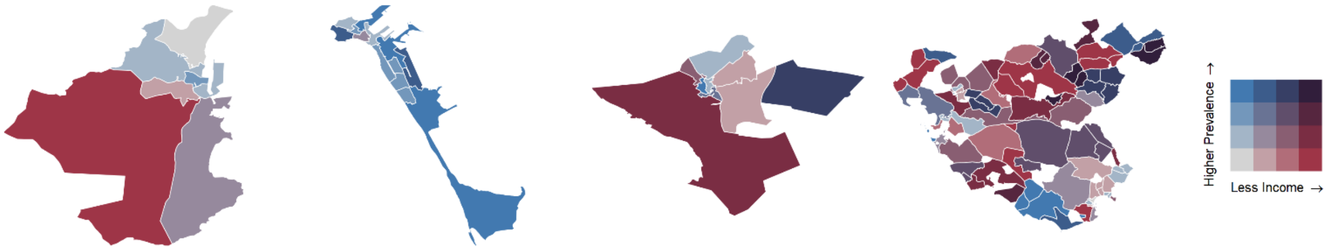

Supplementary Figure 40 | Prevalence of P19: Cardiometabolic + Cardiovascular by local health area. In the maps, we can see the absolute prevalence of the pattern within all persons with multimorbidity at the level of the whole province (right side map) and the level of the three main urban centres of the province (left side of the map).

P20: Dependence + Liver Disease

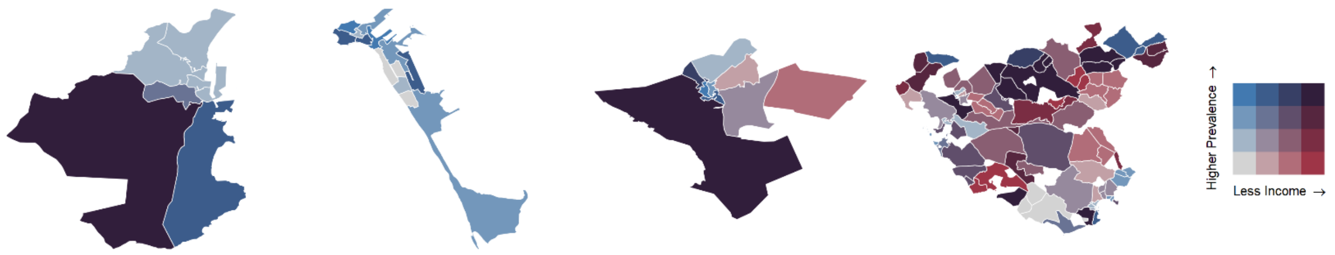

Supplementary Figure 41 | Prevalence of P20: Dependence + Liver Disease by local health area. In the maps, we can see the absolute prevalence of the pattern within all persons with multimorbidity at the level of the whole province (right side map) and the level of the three main urban centres of the province (left side of the map).

P21: Cardiovascular

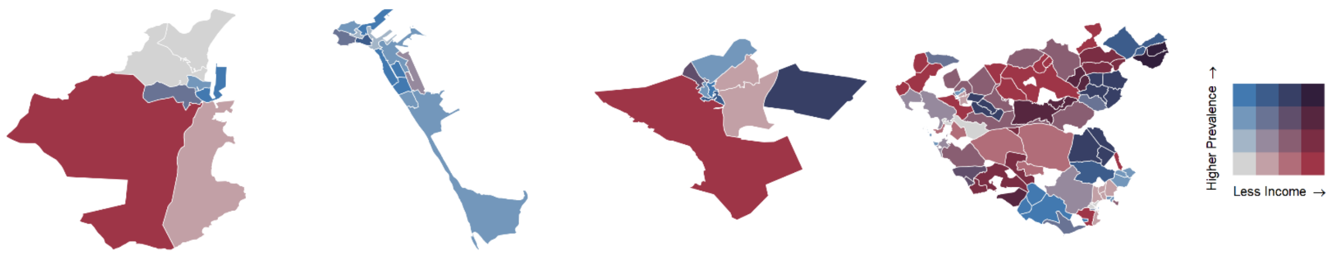

Supplementary Figure 42 | Prevalence of P21: Cardiovascular by local health area. In the maps, we can see the absolute prevalence of the pattern within all persons with multimorbidity at the level of the whole province (right side map) and the level of the three main urban centres of the province (left side of the map).

P22: Cardiometabolic + Cardiovascular + Musculoskeletal + Respiratory (Complex)

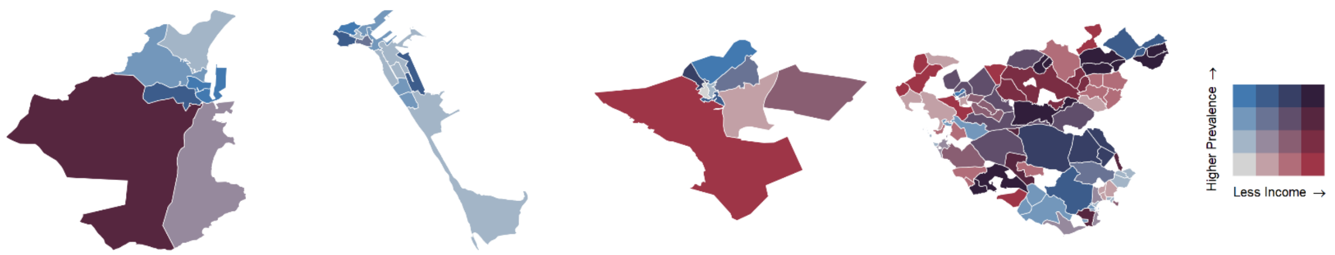

Supplementary Figure 43 | Prevalence of P22: Cardiometabolic + Cardiovascular + Musculoskeletal + Respiratory (Complex) by local health area. In the maps, we can see the absolute prevalence of the pattern within all persons with multimorbidity at the level of the whole province (right side map) and the level of the three main urban centres of the province (left side of the map).

P23: Cardiometabolic + Musculoskeletal

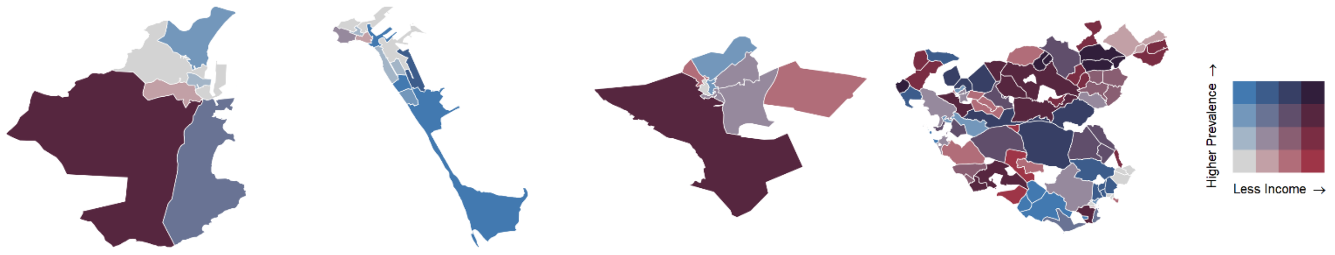

Supplementary Figure 44 | Prevalence of P23: Cardiometabolic + Musculoskeletal by local health area. In the maps, we can see the absolute prevalence of the pattern within all persons with multimorbidity at the level of the whole province (right side map) and the level of the three main urban centres of the province (left side of the map).

P24: Dementia + Mental

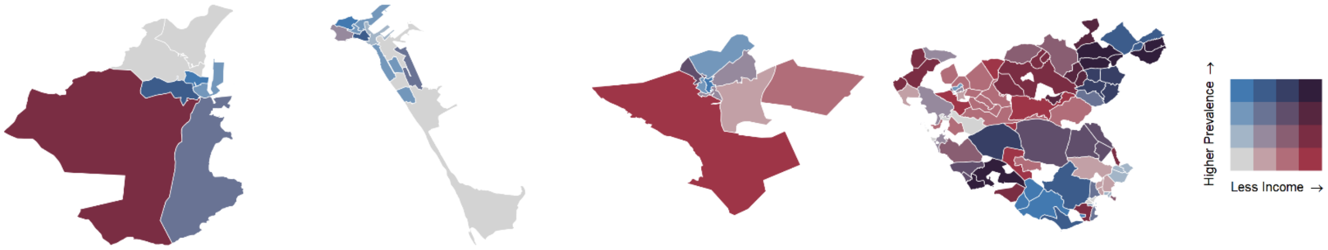

Supplementary Figure 45 | Prevalence of P24: Asthma + Food Intolerance by local health area. In the maps, we can see the absolute prevalence of the pattern within all persons with multimorbidity at the level of the whole province (right side map) and the level of the three main urban centres of the province (left side of the map).

P25: Cardiometabolic + Cardiovascular + Musculoskeletal + Respiratory + Retinopathy + Renal (Complex)

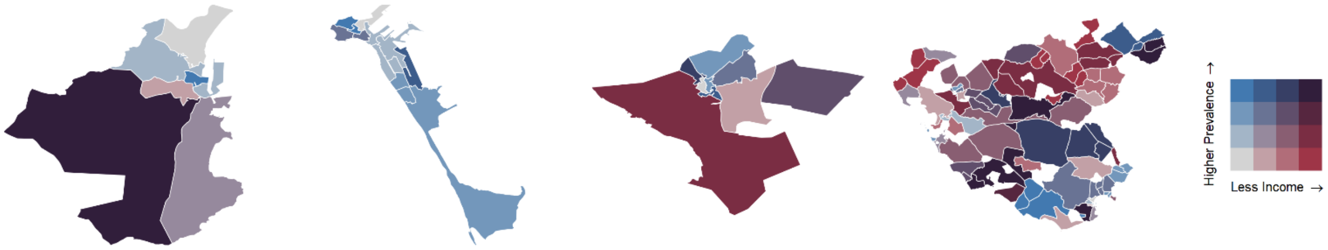

Supplementary Figure 46 | Prevalence of P25: Cardiometabolic + Cardiovascular + Musculoskeletal + Respiratory + Retinopathy + Renal (Complex) by local health area. In the maps, we can see the absolute prevalence of the pattern within all persons with multimorbidity at the level of the whole province (right side map) and the level of the three main urban centres of the province (left side of the map).

4. Supplementary Code Description, ***Characterisation of multimorbidity patterns by local health area in South Spain: epidemiology and impact on the use of health services.***

To obtain the multimorbidity patterns in Cadiz population, we used Latent Class Analysis (LCA), a multivariate technique used to classify observations based on patterns of categorical responses, to identify multimorbidity patterns in the different sex and age subgroups. LCA is a well-suited technique for our data, given the binary variables associated with chronic conditions and the causal relationship we hypothesised between the conditions in each pattern

Given the large sample size and the number of conditions, we computed the LCA models in R with the POLCAParallel package, which implements the usual LCA using all processor cores, thus decreasing computation time. This package allows latent class models with different numbers of classes to be obtained and provides the appropriate fit indices to decide which model has the best characteristics.

The SCRIPT LCA file contains the code to perform the latent class analysis on a simulated sample containing the sex, age, postcode and chronic conditions of 5000 individuals. The sample is simulated due to the protection of the health data used. Since we decided to stratify the sample according to the sex and age of the individuals, the stratification into the 10 age and sex groups is first shown in the code.

Once the 10 groups have been created, we use a for loop and the POLCAParallel package to perform the LCA. With this, we obtain 10 lists containing 11 latent class models ranging from 2 to 12 classes. These models need to be compared to obtain the most suitable model for our data.

```
#LCA Models

lca_2a12 <- list()
for(i in 1:10){
  dat_lca <- dat_multi[[i]][,-ncol(dat_multi[[i]])] +1 #Polca needs 1-2 values
  x <- paste(c("X"), 1:ncol(dat_lca), sep="")
  etiquetas[[i]] <- cbind(x,names(dat_lca))
  colnames(dat_lca)=x
  f <- as.formula(paste("cbind(", paste("X", c(1:ncol(dat_lca)), sep = "", collapse = ","),
")~1", sep ="", collapse=""))
  tic("Latent Class")#To know the computation time
  lca_2a12[[i]]                    <-                    lapply(2:12,                    function(x)
{poLCA(f,dat_lca,nclass=x,maxiter=1500,tol=1e-6,nrep=5,verbose=F)})
  toc()
}
```

We established the number of appropriate patterns in each LCA model, considering three criteria. Initially, we assessed the goodness-of-fit indices of the models, taking into consideration BIC, ABIC, and CAIC. A lower value of these indices indicates a better fit of the model. Additionally, we examined the probability of membership in each class (i.e., multimorbidity pattern) and assessed the clinical interpretability of the results.

To do this, we observe in the code how we make two types of indicative graphs. Firstly, we have a graph in which we review the goodness-of-fit indices (figures S2,S4,S6,S8,S10,S12,S14,S16,S18 and S20), obtained using the Tidyverse environment of R. The observation of these 10 figures allows us to choose which models have the best statistical characteristics.

Once we have chosen the possible LCA models that best fit our data, we must move on to the clinical interpretation phase, in which we will use the second indicative graphs. For this we rely on visually reviewing the prevalences of each chronic condition and their probability of belonging to each multimorbidity pattern, again making use of the Tidyverse work packages. We obtain in the code a list containing 11 figures for each of the 10 groups. We review only those models that present

the best characteristics in the goodness-of-fit indices, allowing us to finally choose the model with the best clinical interpretation (figures S3,S5,S7,S9,S11,S13,S15,S17,S19 and S21). With this, we obtain the final distribution of the multimorbidity pattern in the Cadiz population.

We also highlight the relevance of the SCRIPT MAPS file, through which we obtain the maps of the prevalence of the patterns. We decided that the map should have relevant information for readers who did not know the geographical area of the province of Cadiz, so in addition to the prevalence of the patterns, we added as reference information the level of income per person in each local health area. To do this, we represented the relationship between the prevalence of the resulting patterns and the income per capita in each local health area in the province using a dual chromatic representation of these variables with the biscale package in R.

This package (biscale) allows a dual chromatic representation, indicating on the one hand in which local health area there is a higher prevalence of the pattern and on the other hand which are the areas with the lowest income. Based on the graphics of the ggplot2 package together with its usual function for map representation (geom_sf), biscale allows the dual scale to be used as a fill within the function, incorporating several dual chromatic scales that use two colours to represent each variable and the mixture of these colours to indicate the relationship between the variables. As an example, the following code script:

```
#Maps
    condition <- c("Algeciras","Cádiz","Jerez", “Province”)
    map <- list()
    for(i in 1:4){
    map[[i]] <- ggplot() +
        geom_sf(data = filter(data,area==condition[i]), mapping = aes(fill = bi_class),
            color = "white", size = 0.1, show.legend = FALSE) +
        bi_scale_fill(pal = “DkViolet2”, dim = 4) +
        theme_void()}
```

Given that with the previous code we only obtained individual maps of each zone (Algeciras, Cádiz, Jerez de la Frontera and Province), we relied on the patchwork package to be able to join the different maps of each zone in such a way that we could obtain as much information as possible in the smallest possible space. This package allows us to represent different graphs in matrix form, so that we can place the graphs of our interest in each position. It also allows a great deal of flexibility in deciding the size and relative position of each graph.
