# Supplementary material for: Epidemiology, mortality, and health service use of local-level multimorbidity patterns in South Spain
Source: Nat Commun. 2023 Nov 24;14:7689. doi: 10.1038/s41467-023-43569-5 (PMC10673852; doi:10.1038/s41467-023-43569-5)
Supplement: Supplementary file 3 — Reporting Summary [file 41467_2023_43569_MOESM3_ESM.pdf]

nature portfolio

Corresponding author(s): Javier Alvarez-Galvez

Last updated by author(s): Oct 19, 2023

Reporting Summary

Nature Portfolio wishes to improve the reproducibility of the work that we publish. This form provides structure for consistency and transparency in reporting. For further information on Nature Portfolio policies, see our Editorial Policies and the Editorial Policy Checklist.

Statistics

For all statistical analyses, confirm that the following items are present in the figure legend, table legend, main text, or Methods section.

n/a	Confirmed	
☐	☒	The exact sample size (*n*) for each experimental group/condition, given as a discrete number and unit of measurement
☒	☐	A statement on whether measurements were taken from distinct samples or whether the same sample was measured repeatedly
☐	☒	The statistical test(s) used AND whether they are one- or two-sided<br>*Only common tests should be described solely by name; describe more complex techniques in the Methods section.*
☐	☒	A description of all covariates tested
☒	☐	A description of any assumptions or corrections, such as tests of normality and adjustment for multiple comparisons
☐	☒	A full description of the statistical parameters including central tendency (e.g. means) or other basic estimates (e.g. regression coefficient) AND variation (e.g. standard deviation) or associated estimates of uncertainty (e.g. confidence intervals)
☐	☒	For null hypothesis testing, the test statistic (e.g. *F*, *t*, *r*) with confidence intervals, effect sizes, degrees of freedom and *P* value noted<br>*Give P values as exact values whenever suitable.*
☒	☐	For Bayesian analysis, information on the choice of priors and Markov chain Monte Carlo settings
☒	☐	For hierarchical and complex designs, identification of the appropriate level for tests and full reporting of outcomes
☒	☐	Estimates of effect sizes (e.g. Cohen's *d*, Pearson's *r*), indicating how they were calculated

Our web collection on statistics for biologists contains articles on many of the points above.

Software and code

Policy information about availability of computer code

Data collection	No software was used in the data collection.
Data analysis	The analysis was based on a large set of health records from the Andalusian Health Service, with a total initial dataset of 1,142,367 individuals. Initially, we selected those individuals with multimorbidity (2 or more pathologies on the database). To simplify the analysis and considering the large differences in the prevalence, we stratified the database into groups according to sex and age obtaining 10 groups.<br>Within each stratum, we streamlined the analysis by focusing on the 64 chronic conditions that exceeded a prevalence threshold of 1%. This process resulted in the formation of 10 sex and age groups. The cumulative number of individuals identified with multimorbidity across these groups amounted to 490,130.<br>We used Latent Class Analysis (LCA) to identify multimorbidity patterns in the different sex and age subgroups. We established the number of appropriate patterns in each LCA model, considering three criteria. Initially, we assessed the goodness-of-fit indices of the models, taking into consideration BIC, ABIC, and CAIC. A lower value of these indices indicates a better fit of the model. Additionally, we examined the probability of membership in each class and assessed the clinical interpretability of the results.<br>Following this analysis, we grouped the local health areas according to their socioeconomic status. We obtained three different groups within the 68 local health areas, which we categorised as low SES (low DI and low income), medium SES (medium DI and medium income) and high SES (high DI and high income). With these groupings, we analysed the differences in the prevalence of the 25 patterns in each age and sex stratum through the Kruskal-Wallis Rank Test. We also calculated the average use of health services (GPs visits, hospital consults and emergencies) for each person over the 8 years and we tested the difference in the use of these services within each pattern and each stratum of the local health area through Kruskal-Wallis Rank Test. Finally, we tested the impact of multimorbidity patterns on mortality through a logistic regression analysis, taking mortality as the dependent variable and the multimorbidity pattern of each person, sex, and age as independent variables. We performed this regression analysis separately in each local health area associated with the SES groups.

We conducted all analyses and tables using RStudio and R (4.2.1). We took the standard 0.05 as the significance level and indicated those significance levels lower than 0.1.

For manuscripts utilizing custom algorithms or software that are central to the research but not yet described in published literature, software must be made available to editors and reviewers. We strongly encourage code deposition in a community repository (e.g. GitHub). See the Nature Portfolio guidelines for submitting code & software for further information.

Data

Policy information about availability of data

All manuscripts must include a data availability statement. This statement should provide the following information, where applicable:

- Accession codes, unique identifiers, or web links for publicly available datasets
- A description of any restrictions on data availability
- For clinical datasets or third party data, please ensure that the statement adheres to our policy

In this research, the data have been obtained as part of the DEMMOCAD project after the acceptance of the regional ethical committee. The study data are available under restricted access for patient confidentiality and privacy concerns of the Population Health Database custodians (Andalusian Public Health System, SSPA), access can be obtained upon request to the Director of Health Care and Health Outcomes of the Andalusian Public Health System (SSPA) who can provide the data for the purpose of use in the health and research environment. The researcher must provide the necessary documents accrediting that the project and/or research to be carried out complies with data protection laws and the application will be submitted to a committee of the SSPA, which will decide whether or not to grant access to the data.

Research involving human participants, their data, or biological material

Policy information about studies with human participants or human data. See also policy information about sex, gender (identity/presentation), and sexual orientation and race, ethnicity and racism.

Reporting on sex and gender	The sex of the individual was that associated with the medical records of the Andalusian population database, so the sex was assigned to each individual. For the analyses carried out, and given the differences in the prevalence of chronic conditions according to sex, the groups were segmented according to age and sex, which allowed us to observe that there were significant differences in the distribution of multimorbidity patterns between men and women.
Reporting on race, ethnicity, or other socially relevant groupings	The variables used to determine the grouping of the population were ecological, as no individual socio-economic variables were available beyond sex, age and local health area of residence of each individual. Specifically, the income per person and the deprivation index of each area will be used as variables to determine the socio-economic status of each place of residence.
Population characteristics	The study is based on the entire population of the province of Cádiz. The province of Cadiz is a region in Southern Andalusia (Spain) with a population of around 1,250,000 inhabitants (approximately 15% of the province lives in rural areas). The population has a mean age of 42.7 years, 51.5% of whom are women and with a prevalence of multimorbidity of 42.9%.
Recruitment	Our study uses data from patients attending a health service, so they were not recruited.
Ethics oversight	The study was conducted in accordance with the Declaration of Helsinki and approved by the Andalusian Biomedical Research Ethics Coordinating Committee (Comité Coordinador de Ética de la Investigación Biomédica de Andalucía) on 14 September 2020 (PEIBA:2249-N-19).

Note that full information on the approval of the study protocol must also be provided in the manuscript.

Field-specific reporting

Please select the one below that is the best fit for your research. If you are not sure, read the appropriate sections before making your selection.

☒ Life sciences ☐ Behavioural & social sciences ☐ Ecological, evolutionary & environmental sciences

For a reference copy of the document with all sections, see nature.com/documents/nr-reporting-summary-flat.pdf

Life sciences study design

All studies must disclose on these points even when the disclosure is negative.

Sample size	No sample size calculation was performed, since this is a study with a dataset of the entire population of the province of Cadiz. We only selected the patients having multimorbidity (i.e. 2 or more chronic conditions) among the population.
Data exclusions	We excluded data from individuals whose postcodes did not belong to local health areas in the province of Cadiz. In addition, we only considered those individuals with multimorbidity (i.e. 2 or more chronic conditions).
Replication	Although we provide the codes used in the different analyses carried out, access to the data is restricted by the health authorities of the Andalusian public health system. In this sense, the data would have to be obtained by prior request to the data custodians.

Randomization	Randomization was not needed since we used all the data available of this population.
Blinding	The information was blinded by the data custodians so that all personal patient records were anonymised and confidential to the analysts.

Reporting for specific materials, systems and methods

We require information from authors about some types of materials, experimental systems and methods used in many studies. Here, indicate whether each material, system or method listed is relevant to your study. If you are not sure if a list item applies to your research, read the appropriate section before selecting a response.

Materials & experimental systems

n/a	Involved in the study	
☒	☐	Antibodies
☒	☐	Eukaryotic cell lines
☒	☐	Palaeontology and archaeology
☒	☐	Animals and other organisms
☒	☐	Clinical data
☒	☐	Dual use research of concern
☒	☐	Plants

Methods

n/a	Involved in the study	
☒	☐	ChIP-seq
☒	☐	Flow cytometry
☒	☐	MRI-based neuroimaging
